# Molecular Engineering for Enhancing the Dielectric and Optoelectronic Properties of Antimony Corroles

**DOI:** 10.1002/smsc.202400589

**Published:** 2025-03-04

**Authors:** Tanmoy Pain, Md. Saifuddin, Anshuman Sahoo, Biplab Mahapatra, Subhajit Kar, Rwiddhi Chakraborty, Satyaprasad P. Senanayak, Sanjib Kar

**Affiliations:** ^1^ School of Chemical Sciences National Institute of Science Education and Research (NISER), Bhubaneswar, An OCC of HBNI Jatni 752050 India; ^2^ Centre for Interdisciplinary Sciences National Institute of Science Education and Research Bhubaneswar, Jatni Khurda Odisha 752050 India; ^3^ Nanoelectronics and Device Physics Lab School of Physical Sciences National Institute of Science Education and Research, An OCC of HBNI Jatni 752050 India

**Keywords:** antimony corroles, density functional theory, high dielectric constant material, organic solar cells, photoresponsivity

## Abstract

Herein, the role of molecular engineering on the optoelectronic properties of antimony corroles with two distinct *β*‐substituents and two different antimony oxidation states is studied. Insertion of a strong electron‐withdrawing SCN group on the bi‐pyrrole unit of the corrole increases the molecular dipole moment. Consequently, the dielectric constant is enhanced by up to threefold, reaching a value of 8 for antimony(V) tetra(thiocyano)corrole, significantly higher than any solution‐processable organic semiconductor reported to date. Moreover, this SCN‐substituted molecule also exhibits an increased charge carrier mobility by at least two orders of magnitude. A combination of suitable metallic oxidation state and SCN substitution is crucial in defining absorption, charge carrier mobility, and dielectric constant, all of which impact photovoltaic performance. The fluorescence quantum yield of the champion molecule increases by 300%, and the charge carrier lifetime is extended by twofold, indicating fewer nonradiative recombination pathways or a lower degree of disorder. Consequently, single‐component photodetectors with white light responsivity as high as 10 A W^−1^, ranking among the best in single‐component donor‐based organic semiconductors, and a single‐component solar cell fabricated from antimony(V) tetra(thiocyano)corrole that exhibits an open‐circuit voltage of 0.7 V, at least three times higher than single‐component poly(3‐hexylthiophene) (P3HT)‐based photovoltaic devices, are demonstrated.

## Graphical abstract

Molecular engineering of antimony corroles with various *β*‐substituents and the ability to modulate the oxidation state of the metal ion is employed to demonstrate exceptional optoelectronic properties. The antimony(V) corrole with SCN substituents shows enhanced charge carrier mobility, a high dielectric constant, and a broad absorption range, resulting in superior photoresponse and open‐circuit voltage compared to conventional molecular semiconductors.

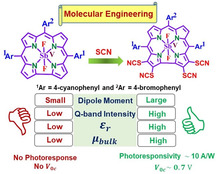

## 1 Introduction

Photovoltaic (PV) research from new‐generation semiconductors has garnered substantial interest in substituting and/or complementing conventional inorganic technologies.^[^ ^1^ ^]^ Among various semiconductors, organic semiconductors (OSCs) have been at the forefront of solution‐processable PV devices. Significant advancement in OPV efficiency has been made through extensive research efforts in device engineering,^[^ ^2^ ^]^ semiconductor microstructure tuning,^[^ ^3^ ^]^ and band structure modification.^[^ ^4^ ^]^ In contrast to other competing technologies, such as inorganic PV or hybrid perovskite photovoltaic devices, where photon absorption leads to free charge carriers, OPV generate bound excitons.^[^ ^5^ ^]^ This is primarily due to low dielectric constant (*ε*_r_), of organic semiconductors which typically ranges from 3 to 5.^[^ ^6^ ^]^ Although a bulk heterojunction can facilitate exciton dissociation, such device structures have associated fabrication challenges, such as solvent compatibility for microstructure control and a limited choice of acceptor molecules.^[^ ^7^ ^]^ This necessitates the development of single‐component optoelectronic devices. It has been projected that when the value of $\varepsilon_{r}$ reaches 10, the efficiencies of OPVs are expected to reach 20% efficiency, provided the charge transport properties remain unaffected.^[^ ^8^ ^]^ Such values of dielectric constant have been observed in inorganic semiconductors and metal halide perovskite semiconductors, which allowed the demonstration of high‐performance PV devices in these materials.^[8b]^ The enhancement in device efficiency can then be attributed to several factors: 1) reduced bimolecular recombination, enabling the fabrication of thicker OPVs for improved light harvesting; 2) reduced exciton binding energy leading to the generation of free carriers; and 3) minimizing the energy offset between singlet and triplet excitons, thereby enhancing the open‐circuit voltage.^[9a]^ Therefore, developing organic semiconducting materials with a high dielectric constant can be a possible strategy to mitigate the major loss factors in OPVs. From a theoretical perspective, a higher dielectric constant can minimize geminate and nongeminate recombination, decrease voltage losses, alleviate space charge effects, and lower exciton binding energy, among other advantages.^[^ ^9^ ^]^ However, in general, an enhancement in dielectric constants is often accompanied by an increase in the material's bandgap due to the associated polarization, as in the case of polymer dielectrics and/or ferroelectric materials.^[^ ^10^ ^]^ This ultimately results in extremely low conductivity. Hence, demonstrating high‐performance OPVs and/or single‐component photodetectors necessitates synthesizing organic semiconducting materials that exhibit a high $\varepsilon_{r}$ value while retaining an appropriate band structure for absorbing the solar spectrum and reasonable bulk charge transport properties.

Various OSCs‐based donors involving porphyrinoids have garnered significant attention due to their exceptional chemical, thermal, and photostability and efficient light‐harvesting capabilities across a large portion of the solar emission spectrum.^[^ ^11^, ^12^, ^13^, ^14^ ^]^ Among the different porphyrinoids, corroles have emerged as one of the most promising candidates for solar energy conversion applications and optoelectronic devices.^[^ ^15^ ^]^ Corroles have one meso‐carbon less than their porphyrin counterparts and are excellent electron donors. Their high charge density in the inner core results in lower oxidation and higher reduction potentials compared to porphyrin analogs.^[^ ^16^ ^]^ Furthermore, their facile one‐step synthesis from commercially available aldehydes and pyrroles presents a cost‐effective alternative to conventional donor OSCs.^[17a]^ Notably, the organometallic complexes of corroles, primarily composed of cyclometalated ligands, have been extensively studied in phosphorescent organic light‐emitting devices due to their naturally long triplet‐excited‐state lifetimes and high‐luminescence quantum yields.^[^ ^18^ ^]^ The extended lifetimes of triplet excitons play a crucial role in reducing exciton recombination, a desirable characteristic for enhancing the performance of OPVs. Furthermore, these complexes typically exhibit broad absorptions throughout the visible spectral range, extending up to 800 nm. This is attributed to the intense mixing between triplet metal‐to‐ligand charge transfer and singlet ligand‐centered π–π* excited states, which proves particularly advantageous for improving OPV performance.^[^ ^19^ ^]^

Despite the ease with which the photophysical properties of metallocorroles can be modified, there are only a few examples of corrole‐based solar cell devices.^[^ ^17^ ^]^ For example, Lai et al. utilized Au corroles to fabricate bulk‐heterojunction solar cell (BHJSC) where three different Au corroles have been investigated as active layers and demonstrated a maximum efficiency of 3.9%.^[17a]^ A gallium corrole linked with BODIPY (Ga‐BODIPY) dyad has been used as an electron donor in a BHJSC, where the integration of corrole and BODIPY dyes allowed for broader absorption of the white light spectrum with high molar extinction coefficients.^[18a]^ The study demonstrated that solvent vapor annealing treatment can improve the morphology of the film, which in turn enhances the exciton generation and the consequent dissociation in free charge carriers, leading to the highest photoconversion efficiency of 6.6%. In addition to the usage of Corrole‐based semiconductors as active semiconducting layer, Cu corroles have also been used as a hole transporting layer (HTL) in a perovskite solar cells.^[19a]^ Owing to the higher thermal stability of these corrole‐based compounds in comparison to the conventional HTL, the devices exhibited enhanced stability to extended thermal stress at 85 °C. Notably, no prior reports on the PV performance of antimony corroles have been found in the literature, and more critically, a general understanding which correlates molecular design with the PV device performance is necessary to further advance facile tunability of dielectric and optoelectronic properties, enabling realization of the potential of these class of semiconductors in devices.

**Scheme 1:**
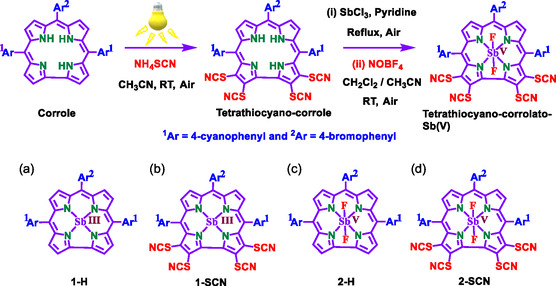
The chemical structure of a) {10‐(4‐bromophenyl)‐5,15‐bis(4‐cyanophenyl) corrolato}antimony(III), antimony(III) corrole, **1‐H**; b) {2,3,17,18‐*tetra*(thiocyano)‐10‐(4‐bromophenyl)‐5,15‐bis(4‐cyanophenyl) corrolato} antimony(III), antimony(III) corrole, **1‐SCN**;^[^ ^20^ ^]^ c) {10‐(4‐bromophenyl)‐5,15‐bis(4‐cyanophenyl) corrolato} (*trans*‐difluoro) antimony(V), antimony(V) corrole, **2‐H**. d) {2,3,17,18‐*tetra*(thiocyano)‐10‐(4‐bromophenyl)‐5,15‐bis(4‐cyanophenyl) corrolato} (*trans*‐difluoro) antimony(V), antimony(V) corrole, **2‐SCN**.

In order to highlight the impact of molecular engineering on the optoelectronic characteristics of antimony corrole, we present and compare three novel antimony corrole complexes as well as one that was previously reported in the literature: {10‐(4‐bromophenyl)‐5,15‐bis(4‐cyanophenyl)corrolato}antimony(III) (referred to as antimony(III) corrole, **1‐H**), {2,3,17,18‐*tetra*(thiocyano)‐10‐(4‐bromophenyl)‐5,15‐bis(4‐cyanophenyl)corrolato}antimony(III) (antimony(III) corrole, **1‐SCN**),^[^ ^20^ ^]^ {10‐(4‐bromophenyl)‐5,15‐bis(4‐cyanophenyl)corrolato}*trans*‐difluoro antimony(V) (antimony(V) corrole, **2‐H**), and {2,3,17,18‐*tetra*(thiocyano)‐10‐(4‐bromophenyl)‐5,15‐bis(4‐cyanophenyl)corrolato}*trans*‐difluoro antimony(V) (antimony(V) corrole, **2‐SCN**) (**Scheme** **1**).

These compounds can be categorized as having two distinct *β*‐substituents and two different antimony oxidation states. The strategy for fine tuning the optical and charge carrier properties of antimony porphyrinoids involved inserting four SCN‐substituents at the 2,3,17, and 18 *β*‐pyrrolic positions of the corrole, a careful selection of the oxidation state of antimony, and the addition of two *trans*‐axial fluoride ligands. In contrast to earlier studies that largely centered on porphyrinoids without extensive exploration of systematic molecular modifications, this work introduces a unique strategy by regioselectively incorporating four strongly electron‐withdrawing SCN groups. This approach significantly improves the absorption characteristics of Q‐bands compared to conventional porphyrins and corroles. The interplay between the oxidation state and SCN substituents is pivotal in shaping absorption properties, charge carrier mobility, and achieving a high dielectric constant, all of which are essential for enhancing PV performance. We present a comprehensive molecular understanding of various optoelectronic characteristics through a combination of optical spectroscopy, electrical charge transport, and impedance measurement. Antimony's oxidation state primarily influences its optical absorption, with lower oxidation states producing redshifted absorption spectra. On the other hand, incorporating SCN substituent results in higher intensity of Q‐band absorption, enhanced charge carrier mobility, and high dielectric constant, all of which significantly impact their PV performance. From DFT calculation, we obtain that the higher polarizability is attributed to the dissimilar parity and spatial coexistence of the charge density between the two wave functions in these molecules, which, however, has no effect on the charge transport.^[^ ^21^ ^]^ Owing to this outstanding combination of higher polarization, enhanced charge mobility, and broad visible absorption, we demonstrate a single‐component photodetector and solar cell with white light photoresponsivity of up to 10 A W^−1^ and open‐circuit voltage three times greater than the commercial polymer.

## 2 Results and Discussion

### 2.1 Synthesis and Characterization

The FB {2,3,17,18‐tetra(thiocyano)‐10‐(4‐bromophenyl)‐5,15‐bis(4‐cyanophenyl) corrole was synthesized using a previously reported method.^[^ ^22^ ^]^ The antimony(III) corrole, **1‐H**, can be prepared by refluxing a mixture of the free base corrole and SbCl_3_ for several hours.^[23h]^ Treating the antimony(III) corrole, **1‐H** with an excess of NOBF_4_ in a CH_2_Cl_2_—CH_3_CN mixture resulted in the formation of *trans*‐difluoroantimony(V) {2,3,17,18‐tetra(thiocyano)‐corrole derivative, antimony(V) corrole, **2‐SCN** (Scheme 1). Similarly, treating the antimony(III) corrole **1‐H**, with an excess of HF in CH_2_Cl_2_ resulted in the formation of a *β*‐unsubstituted *trans*‐difluoroantimony(V) corrole (antimony(V) corrole, **2‐H).** The composition and purity of the antimony corroles were confirmed through a series of analytical techniques, including CHN analyses, UV/Vis, Fourier transform infrared spectroscopy, ESI‐MS, NMR, and single‐crystal X‐ray diffraction (SC‐XRD) analysis (Table S1–S3 and Figure S1–S18, Supporting Information). The ^1^H NMR spectra of antimony(III) corrole, **1‐H** and antimony(V) corrole, **2‐SCN** display intense peaks in the range of ≈9.26–7.89 ppm and ≈8.84–7.97 ppm, respectively (Figure S3 and S5, Supporting Information). The FT‐IR spectra (as KBr pellets) of antimony(V) corrole, **2‐SCN**, showed a strong S‐CN stretching vibration at 2160 cm^−1^ (Figure S2, Supporting Information). The electrospray mass spectrum of antimony(III) corrole, **1‐H**, in acetonitrile exhibited peaks centered at *m/z* = 771.9943, corresponding to [**M**]^+^ (771.9971 calculated for C_39_H_20_BrN_6_Sb), and for antimony(V) corrole, **2‐SCN** at *m/z* = 1060.8589, corresponding to [**M** + Na]^+^ (1060.8524 calculated for C_43_H_16_BrN_10_F_2_S_4_SbNa) (Figure S8 and S9, Supporting Information). Conductivity data indicate the absence of dissociable counter ions in these solutions.

### 2.2 Crystal Structure

**Figure 1:**
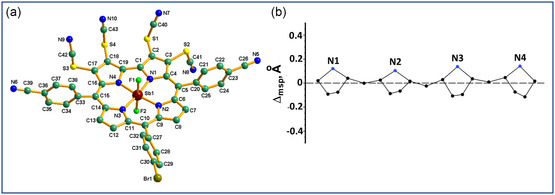
a) Single‐crystal X‐Ray structure, and b) linear display of nonplanar distortions for antimony(V) corrole, **2‐SCN**. Hydrogen atoms are omitted for clarity. Distances (Å): Sb1‐N1 1.993(6), Sb1‐N2 1.982(7), Sb1‐N3 1.971(7), Sb1‐N4 1.985(6), Sb1‐F1 1.923(5), Sb1‐F2 1.936(5).

The crystal structure of antimony(V) corrole, **2‐SCN**, is depicted in **Figure** **1**a. Key crystallographic parameters for antimony(V) corrole, **2‐SCN**, are summarized in Table S1, Supporting Information. It adopts a monoclinic structure, with each unit cell containing four antimony(V) corrole molecules, **2‐SCN**. The N1‐Sb‐N2, N2‐Sb‐N3, N3‐Sb‐N4, and N4‐Sb‐N1 bite angles for antimony(V) corrole, **2‐SCN**, measure 92.3(3)° (DFT: 92.2°), 96.0(3)° (DFT: 95.9°), 91.9(3)° (DFT: 92.1°), and 79.8(3)° (DFT: 79.7°), respectively. The Sb—N bond distances are 1.993(6) Å (DFT: 2.009 Å) for Sb—N1, 1.982(7) Å (DFT: 1.983 Å) for Sb—N2, 1.971(7) Å (DFT: 1.985 Å) for Sb—N3, and 1.985(6) Å (DFT: 2.004 Å) for Sb—N4. The Sb—F bond distances are 1.923(5) Å (DFT: 1.893 Å) for Sb—F1 and 1.936(5) Å (DFT: 1.896 Å) for Sb—F2. The F1–Sb1–F2 angle is 178.0(2)° (DFT: 179.8°). These bond distances and angles are consistent with known antimony(V) corroles.^[23h]^ A PorphyStruct analysis of antimony(V) corrole, **2‐SCN**, is presented in Figure 1b.^[^ ^24^ ^]^ Antimony(V) corrole, **2‐SCN**, exhibits an overall distortion (D_oop_) of 0.3710 Å. The primary modes of out‐of‐plane deformation include a doming distortion of 0.3662 Å (accounting for 69.7% of the total distortion), a ruffling distortion of 0.0529 Å (10.1% of the total distortion), a saddling distortion of ‐0.0084 Å (1.6% of the total distortion), and a total waving distortion ranging from −0.0577 to 0.0015 Å (18.7% of the total distortion).

The Hirshfeld surfaces analysis^[25a]^ of antimony(V) corrole, **2‐SCN**, was conducted to identify the weak interactions within its crystal lattice. Close contacts such as N⋯H, H⋯H, and C⋯H were observed in higher percentages than weaker interactions like N⋯C, F⋯H, and S⋯H, among others, in the crystal lattice of antimony(V) corrole, **2‐SCN** (see Table S3 and Figure S10–S12, Supporting Information). SC‐XRD analysis underscores the presence of noncovalent chalcogen bonds, particularly S···N≡C interactions, within the solid state (Figure S11, Supporting Information). These interactions lead to intermolecular face‐to‐face stacking of molecules, resulting in a well‐ordered configuration with uniform spacing. The S···N≡C contact distance measures 3.05 Å in **2‐SCN**, which is less than the combined van der Waals radii of 3.35 Å for S···N. The angle between the S–N–C atomic units in these interactions is 147.5°. Chalcogen bonds are vital for defining the secondary coordination sphere in coordination compounds, significantly affecting the development of various supramolecular architectures.^[25b–d]^

### 2.3 UV–Vis and Emission Spectroscopy

**Figure 2:**
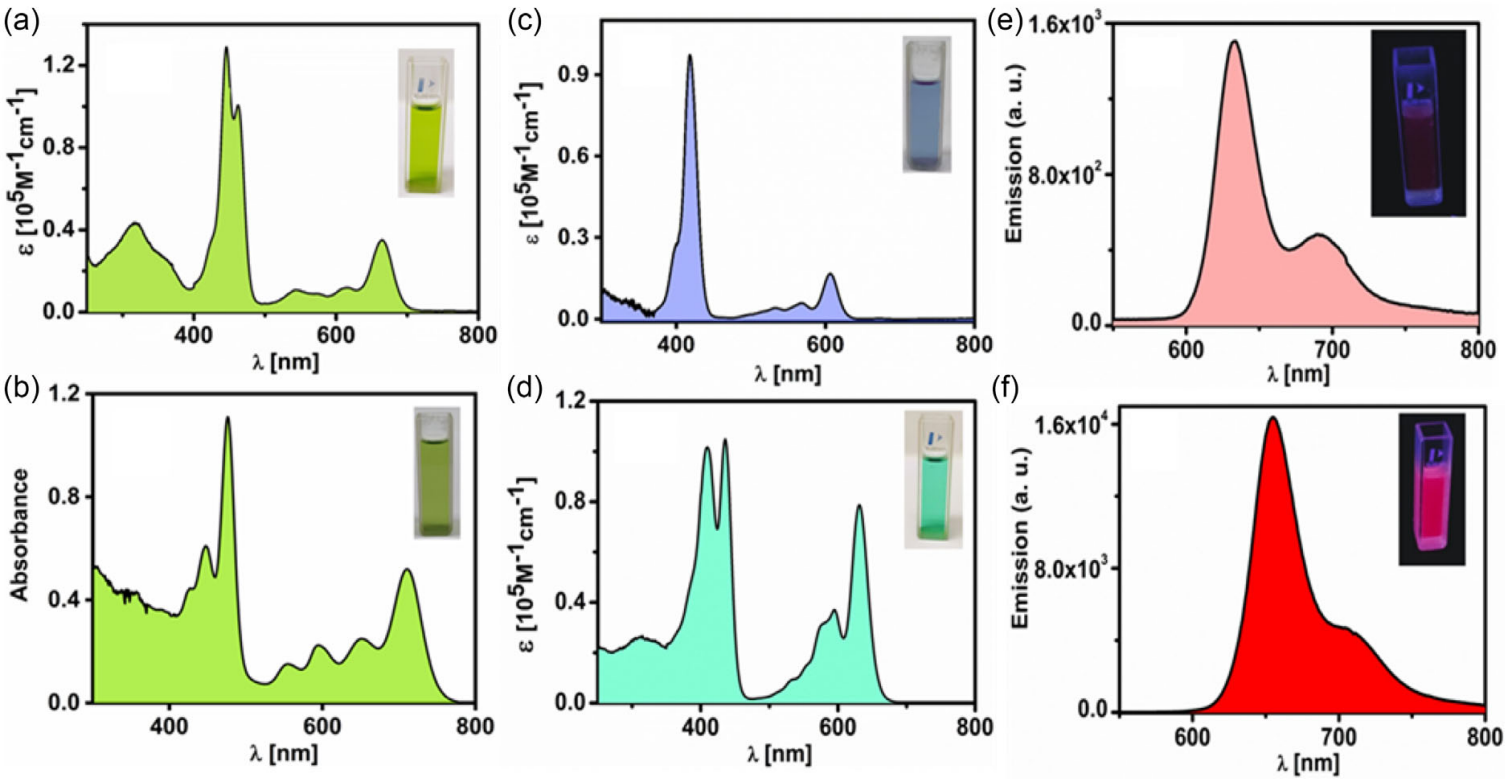
Steady‐state absorption of antimony(III) corrole, a) **1‐H**, antimony(III) corrole, b) **1‐SCN**, antimony(V) corrole, c) **2‐H**, and antimony(V) corrole d) **2‐SCN**. Emission spectra of antimony(III) corrole, e) **1‐H** (λ_exc_ = 446 nm), and antimony(V) corrole f) **2‐SCN** (λ_exc_ = 410 nm) in solution.

**Figure 3:**
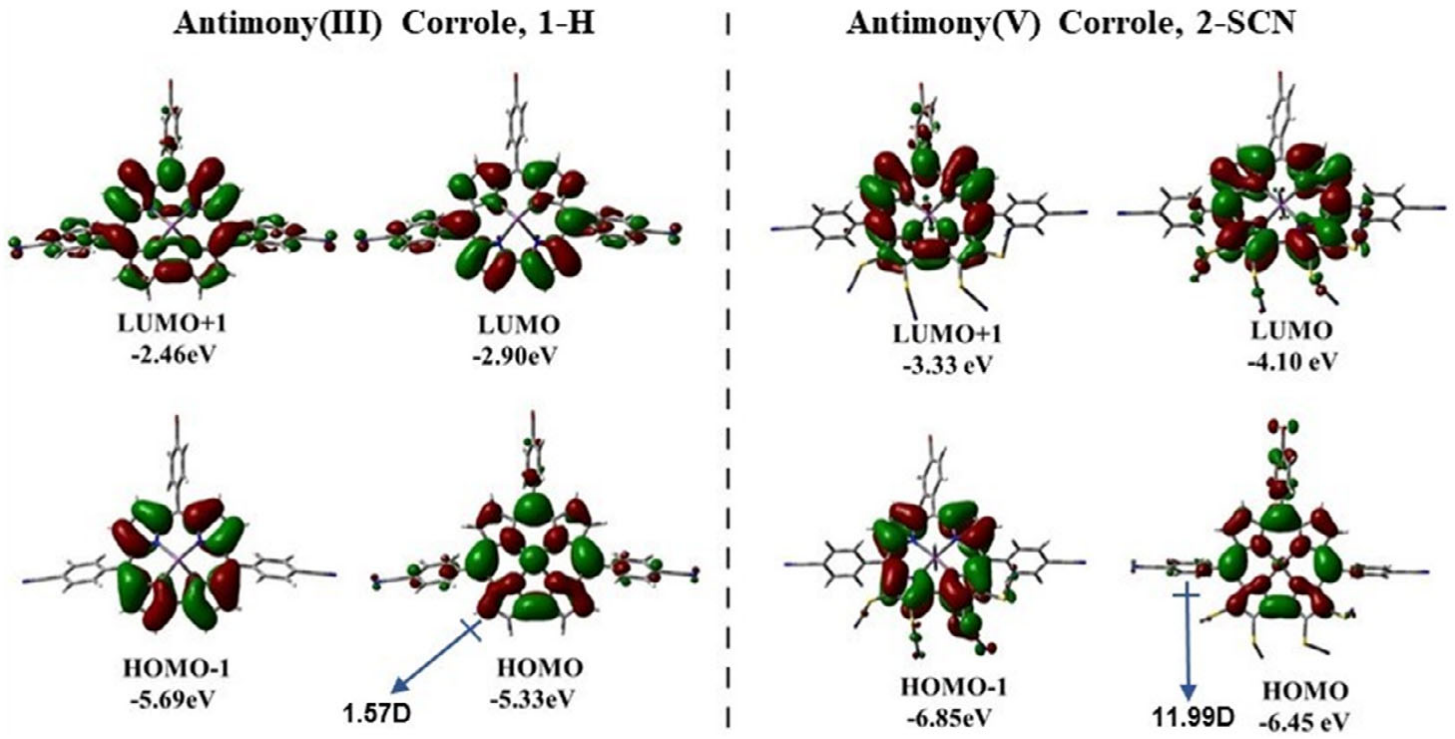
Selected frontier MOs and their orbital energies of antimony(III) corrole, **1‐H**, and antimony(V) corrole, **2‐SCN**. The orbitals that contribute to the polarity of the molecules are depicted.

The electronic absorption spectra of all the corrole molecules in the solution are displayed in **Figure** **2**a–d (Table S2, Supporting Information). In general, antimony(III) corrole, **1‐H**, exhibits a split Soret‐type band over a range of 446–462 nm, and Q‐type bands are observed in the range of 530–670 nm, which are characteristic of antimony(III) corroles. The molar absorption coefficient of the Q‐type bands for antimony(III) corrole, **1‐H**, falls within the range of 10–35 × 10^3^ M^−1^ cm^−1^. The intense Soret‐type band at 446 and 462 nm arises from a combination of transitions between HOMO‐1 → LUMO, HOMO → LUMO + 1, HOMO → LUMO + 2, HOMO‐1 → LUMO + 1, and HOMO → LUMO + 3 orbitals (Highest Occupied Molecular Orbital (HOMO) and Lowest Unoccupied Molecular Orbital (LUMO); Table S4, Supporting Information). Nevertheless, the main transitions of antimony(III) corrole, **1‐H**, can be mainly attributed to the ligand‐based frontier orbitals (**Figure** **3**). Furthermore, in the antimony(III) corrole, **1‐H**, both the HOMO‐3 and HOMO orbitals exhibit contributions from the lone pair. This lone pair possibly leads to the quenching of the excited state and a reduction in fluorescence quantum yield.^[23k]^ Upon SCN substitution in place of H in antimony(III) corrole, **1**, there is an intensity enhancement and redshift for Q band absorption.

On the other hand, antimony(V) corrole, **2‐SCN**, also displays a split Soret‐type band at 410 and 435 nm. The molar absorption coefficient of the Soret bands for compound **2** falls within the range of 1.01–1.05 × 10^5^ M^−1^ cm^−1^. Antimony(V) corrole, **2‐SCN**, exhibits a strong Q peak at 631 nm with a molar absorption coefficient of 0.8 × 10^5^ M^−1^ cm^−1^. Therefore, in antimony(V) corrole, **2‐SCN**, the intensities of the Soret and Q bands are nearly equal, which is an unusual occurrence in normal porphyrins and corroles. The intense Soret‐type band at 410 and 435 nm results from a combination of transitions between HOMO‐2 → LUMO + 1, HOMO‐1 → LUMO, HOMO → LUMO + 1, and HOMO‐1 → LUMO + 1 orbitals (Figure 3 and Table S5, Supporting Information). These orbitals are exclusively located on the corrole ring. The calculated UV−vis spectra of both the antimony corroles, using time‐dependent density functional theory (TD‐DFT) calculations, show a good correlation with the observed absorption profile (Table S4 and S5, Figure S19–S22, Supporting Information). Similar to other *tetra*‐SCN‐substituted metallocorroles, the strong and redshifted Q band is attributed to the reduction in macrocycle aromaticity due to thiocyanation, the narrowing of the HOMO–LUMO gap, and the widening of the LUMO/LUMO + 1 gap.^[^ ^26^ ^]^ Notably, analysis of the optimized structures highlighted a larger skeletal bond distance alternation in **2‐SCN** compared to *β*‐unsubstituted antimony(V) corrole, which is consistent with the previously mentioned crystallographic results.^[^ ^26^ ^]^ These calculations were performed using the B3LYP level of theory along with 6‐G11 (d, p) basis sets. DFT and TD‐DFT calculations offer a cohesive explanation for several of the experimental findings discussed here. The optimized geometries of antimony(III) corrole, **1‐H**, and antimony (V) corrole, **2‐SCN**, strongly aligned with experimental data. Specifically, the calculated Sb—N bond lengths in antimony(V) corrole, **2‐SCN**, matched the experimental values almost exactly. Furthermore, the calculations corroborated the slight saddling of the corrole macrocycle. H substitution at *β*‐position in place of SCN in antimony(III) corrole results in an intensity decrease and blueshift for Q band absorption. Overall upon comparing antimony corrole, we conclude that corroles having higher oxidation states exhibit blue shifted absorption, whereas SCN substituent corroles show higher Q band intensity when compared to their counterpart.

The absorption spectra of thin films (Figure S18, Supporting Information) of macrocycles exhibit a distinct redshift and broadening of the Soret and Q‐bands, attributable to the orientation of the transition dipole and excitonic coupling, which is commonly observed in porphyrinoid macrocycles.^[^ ^27^ ^]^

For comparison only the emission spectra of antimony(III) corrole, **1‐H**, and antimony(V) corrole, **2‐SCN**, are illustrated in Figure 2e,f. In the CH_2_Cl_2_ solution (excited at the Soret band), both complexes exhibit strong peaks at 633 and 654 nm for antimony(III) corrole, **1‐H**, and antimony(V) corrole, **2‐SCN**, respectively. These emissions are identified as fluorescence, as the observed Stokes shifts are relatively small compared to the Q‐type bands. The fluorescence quantum yield (*φ*) values for antimony(III) corrole, **1‐H**, and antimony(V) corrole, **2‐SCN**, in toluene are 0.014% and 3.28%, respectively. The substantial increase in *φ*‐values for antimony(V) corrole, **2‐SCN**, suggests a lower occurrence of nonradiative recombination pathways, indicating lower disorder or density of trap states than antimony(III) corrole, **1‐H**. Due to antimony's efficient spin‐orbit coupling, numerous triplet states are formed in the antimony(III/V) corrole complexes.^[23j]^ Singlet oxygen is often generated via quenching of the triplet excited state. The quantum yields of singlet oxygen production for antimony(III) corrole, **1‐H**, and antimony(V) corrole, **2‐SCN**, were ≈20% and 50% in the CCl_4_ solution, respectively. While direct measurement of triplet lifetime is challenging, earlier reports indicate that the triplet lifetime of antimony(V) corrole, **2‐SCN**, is significantly longer than that of antimony(III) corrole, **1‐H**.^[23k]^ Trap density is directly influenced by the excited state lifetime. Long‐lived excited states are predicted to extend the exciton diffusion length, a crucial factor for enhancing the short‐circuit current in OPVs.^[^ ^28^ ^]^

### 2.4 Redox Properties

The redox properties of both the antimony corroles were investigated in CH_2_Cl_2_ with 0.1 M, TBAP, utilizing cyclic voltammetric and differential pulse voltammogram techniques (Figure S13 and S14, Supporting Information). Antimony(III) corrole, **1‐H**, displayed two one‐electron reversible oxidative peaks at +0.53 and +1.06 V. Additionally, it exhibited one‐quasireversible reductive peak at −1.10 V versus Ag‐AgCl. On the other hand, antimony(V) corrole, **2‐SCN**, demonstrated a one‐electron reversible reductive peak at −0.23 V versus Ag–AgCl. The redox potentials are significantly upshifted compared to the nonthiocyanate complexes,^[23h]^ consistent with the strong electron‐withdrawing character of the four SCN substituents at the β‐pyrrolic positions. The electrochemical E_HOMO_ (energy of the highest occupied molecular orbital) and E_LUMO_ (energy of the lowest unoccupied molecular orbital) were determined from the cyclic voltammograms (CVs) of both the antimony corroles. Antimony(III) corrole, **1‐H**, exhibited a LUMO level of −3.2 eV and a HOMO energy level of −5.3 eV through cyclic voltammetry measurements. Meanwhile, antimony(V) corrole, **2‐SCN** displayed a LUMO level of −4.1 eV and a HOMO energy level of −6.0 eV.

### 2.5 Dielectric Properties

**Figure 4:**
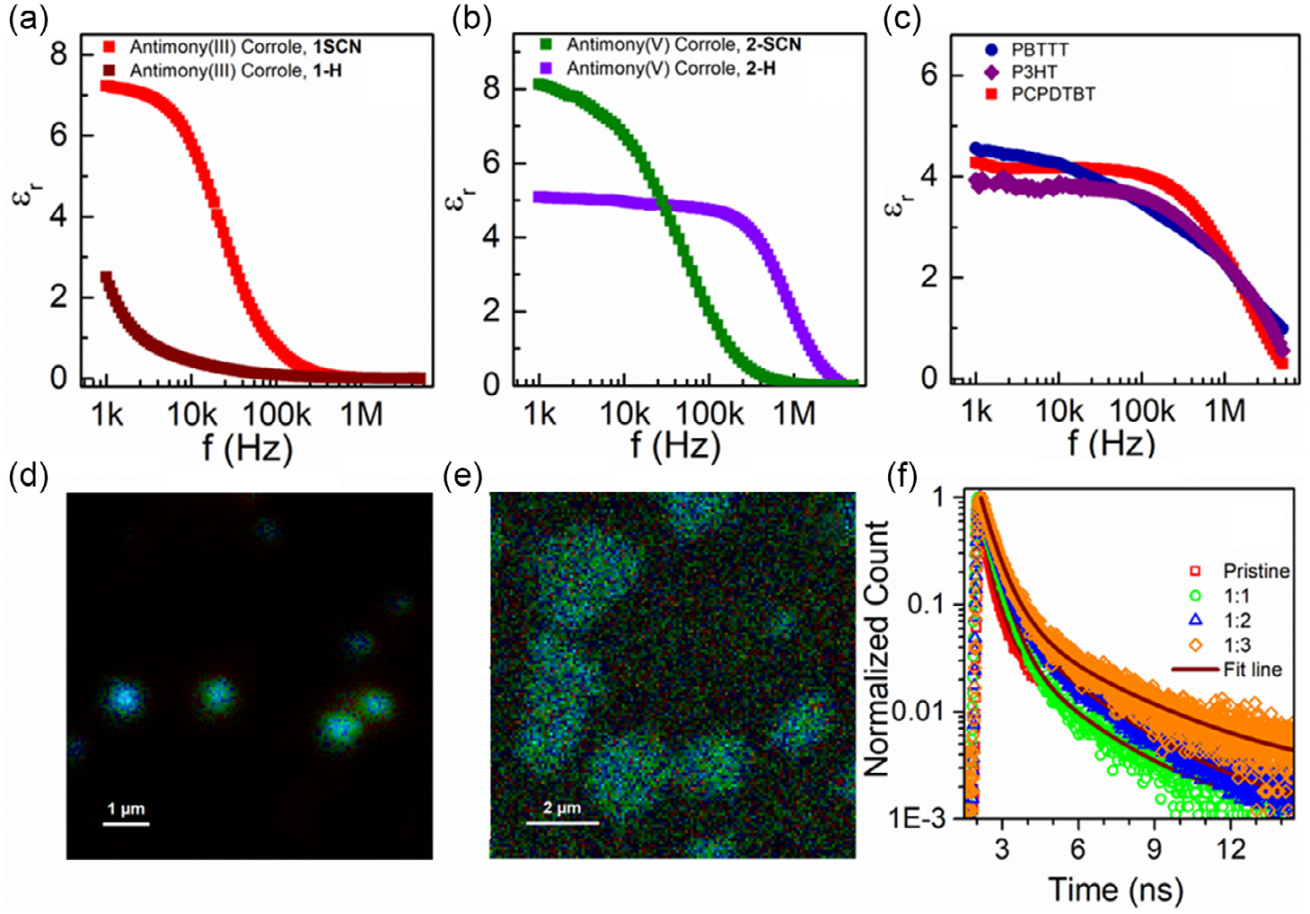
Impedance measurement plot of a) antimony(III) corrole, **1‐H**, and antimony(III) corrole, **1‐SCN**; b) antimony(V) corrole, **2‐H**, and antimony(V) corrole, **2‐ SCN**; c) various other commercially available polymers which are used in PV devices. FLIM for d) pristine film of antimony(V) corrole, **2‐SCN**; e) blended film of PVDF: HFP with antimony(V) corrole, **2‐SCN**, in ratio 1:3. f) The plot of the fluorescence lifetime for films with a different fraction of PVDF: HFP incorporated into antimony(V) corrole, **2‐SCN**.

Designing an efficient OSC for PV devices necessitates a broad absorption window to maximize solar spectrum utilization and facilitate the generation of excitons (bound electron–hole pairs). Furthermore, a higher dielectric constant (*ε*_r_) is essential to facilitate the dissociation of excitons into free charge carriers. Therefore, detailed capacitance measurements were conducted for both antimony corroles using a sandwich device structure. **Figure** **4**a,b presents the $\varepsilon_{r}$–*f* curves for antimony corrole(III), **1**, and antimony corrole(V), **2**, with their respective *β*‐substituents (SCN or H). SCN substitution of antimony corrole(III), **1**, increases the $\varepsilon_{r}$‐value from 2.5 to 7.2 at *f* = 1 kHz, whereas H substitution in place of SCN for antimony corrole(V), **2**, reduces the $\varepsilon_{r}$‐value from 8.1 to 5.1 at *f* = 1 kHz. Thus, we conclude that irrespective of the oxidation state, SCN substitution in antimony corrole results in a higher dielectric constant ($\varepsilon_{r}$ ≈ 7–8), whereas H substitution at the *β*‐position leads to lower $\varepsilon_{r}$ values. This underscores the significance of SCN substitution in determining the dielectric properties of antimony corroles. Further, to elucidate the role of molecular design, we performed detailed impedance measurements on other commercial donor molecules, as shown in Figure 4c, where the $\varepsilon_{r}$‐values range between 3.9 and 4.6. This is consistent with the report by Torabi et al. which shows that $\varepsilon_{r}$ values for most donor, acceptor, and donor–acceptor‐type OSCs typically range between 3 and 6.^[^ ^29^ ^]^ Hence, it should be noted that the incorporation of SCN groups on the bipyrrole unit of the corrole significantly enhances the $\varepsilon_{r}$ value of antimony(V) corrole **2‐SCN**, making it the highest among majority of organic semiconductors.

Based on a typical Columbic interaction, the exciton binding energy ($E_{\text{exc}}$) is inversely proportional to the $\varepsilon_{r}$ value of an organic molecule ($E_{\text{exc}}=\frac{e^{2}}{4\pi \varepsilon_{r}\varepsilon_{0}R_{0}}$, where *e* is the electronic charge and $R_{0}$ exciton radius).^[^ ^30^ ^]^

Considering $R_{0}=4$nm,^[^ ^30^ ^]^ the $E_{\text{exc}}$ values for two extremes antimony(III) corrole, **1‐H**, and antimony(V) corrole, **2‐SCN**, were estimated to be ≈144 and ≈44 meV, respectively. Hence, for antimony(V) corrole, **2‐SCN**, excitons can easily dissociate into free charge carriers, ensuring efficient device performance (details provided in a later section). Furthermore, to verify our experimental observations regarding the role of SCN, we performed DFT calculations for two extreme molecules with different oxidation states of antimony and *β*‐substituents: antimony(III) corrole, **1‐H**, and antimony(V) corrole, **2‐SCN**. Interestingly, the DFT‐calculated molecular dipole moment of antimony(V) corrole, **2‐SCN**, is found to be 11.99 D, significantly larger than that of antimony(III) corrole, **1‐H** (dipole moment ≈ 1.57 D) (Table S6, Supporting Information). To further understand the origin of polarization, we estimated the quadrupole moment of antimony(V) corrole, **2‐SCN**. The quadrupole moment measures the charge distribution within the molecule. From the DFT calculations, we obtained a large quadrupole moment of −408.98 Debye·Å for antimony(V) corrole, **2‐SCN**, which can be attributed to the electron‐withdrawing effect of the four SCN groups (for SCN, Hammett's *σ*_p_ = 0.52 and *σ*_m_ = 0.51). The multiple thiocyanate groups are arranged in close proximity and oriented in a specific manner within the molecule, allowing their dipole moments to combine or reinforce each other, resulting in an overall higher dipole moment for the entire molecule. In summary, from both the capacitance measurements and DFT calculations, we observe that we can significantly enhance molecular polarization in OSCs with appropriate molecular engineering (in this case, SCN substitution). To directly visualize the effect of dielectric constant variation on the fluorescence properties, we conducted detailed fluorescence lifetime imaging (FLIM) of films fabricated by blending the corrole molecules with the dielectric material PVDF: HFP at varied concentrations (details provided in the method section, Supporting Information). Figure 4d,e, S23, and S24, Supporting Information, show the FLIM of the thin films fabricated from these blends. A clear enhancement in fluorescence is observed upon increasing the PVDF: HFP concentration. We then fitted the spatial distribution of the FLIM with a Gaussian function to estimate the full width at half maximum (FWHM) values (Figure S25 and S26, Supporting Information). The FWHM value for antimony(V) corrole, **2‐SCN**, is marginally lower compared to that of antimony(III) corrole, **1‐H**, suggesting better uniformity and phase purity of the films. Nevertheless, the fluorescence lifetime increases for both antimony corrole molecules upon the incorporation of PVDF:HFP. In the case of antimony(V) corrole, **2‐SCN**, the fluorescence lifetime increases from 419 ps for the pristine material to 803 ps when the PVDF:HFP concentration is tripled (Figure 4f). Similarly, the fluorescence lifetime of antimony(III) corrole, **1‐H**, increases from 203 to 642 ps (Figure S24e, Supporting Information).

To understand the observed enhancement in fluorescence, we performed detailed impedance measurements of these samples. The results indicate that the dielectric constant of the corrole PVDF: HFP blend increases as the concentration of PVDF: HFP increases (Figure S27, Supporting Information), suggesting a direct correlation between the fluorescence lifetime and the dielectric constant. An increase in the dielectric constant of the semiconductor reduces the exciton binding energy ($E_{\text{exc}}$), leading to the creation of free charge carriers and consequently increasing the lifetime of these carriers.

### 2.6 Charge‐Carrier Transport Properties

As a next step, we evaluated the bulk charge transport properties of the synthesized antimony corrole molecules. To determine the charge carrier mobility (*μ*), we employed the space–charge‐limited current (SCLC) technique, which is one of the most reliable methods for investigating a semiconductor's intrinsic transport characteristics and trap density.^[^ ^19^ ^]^ Furthermore, the device structure used for SCLC measurements is similar to that employed in OPVs, where vertical charge transport is predominant. The SCLC method involves measuring the current–voltage (*I–V*) characteristics of a single‐carrier device, in which the metal contacts on both sides of the semiconductor are aligned with the conduction (or valence) band, allowing only electrons (or holes) to be injected and transported efficiently. Hole‐only devices were fabricated with the configuration ITO/PEDOT: PSS/antimony corroles/Au (see Experimental Details, Supporting Information).^[^ ^31^ ^]^

The charge carrier mobility in thin films can be determined by fitting the *J–V* curves in the SCLC region using the equation: $J=\frac{9}{8}\varepsilon_{0}\varepsilon_{r}\mu V^{2}/d^{3}$, where *J* is the current density in the SCLC region, *V* is the applied voltage, $\varepsilon_{0}$ is the permittivity of free space, $\varepsilon_{r}$ is the relative permittivity (dielectric constant) of the thin film, *μ* is the charge carrier mobility, and *d* is the thickness of the thin film.

**Figure 5:**
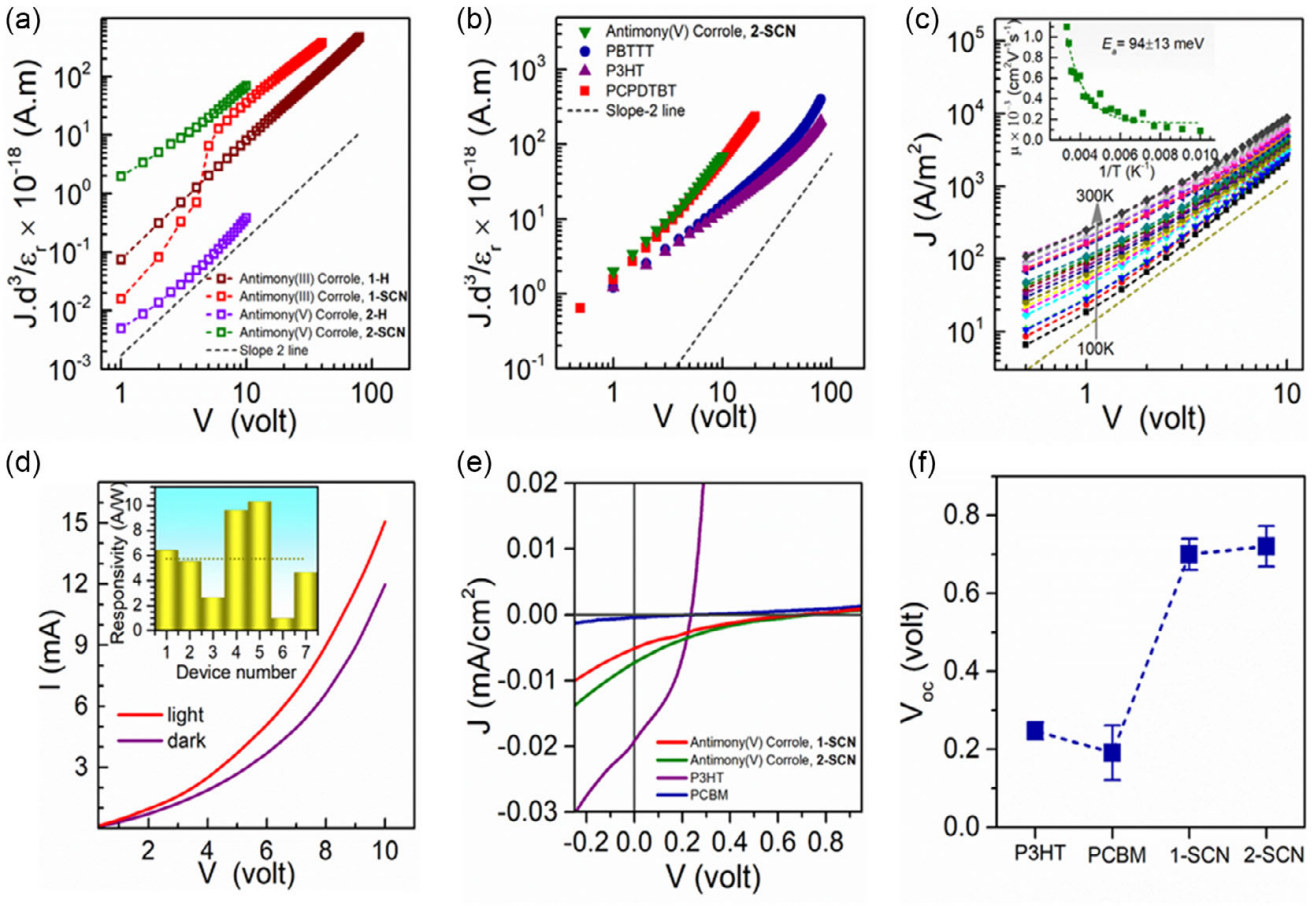
a) *J–V* characteristics of hole‐only devices of corrole molecules at 300 K (*J* is scaled with sample thickness (*d*) and dielectric constant ($\varepsilon_{r}$)). b) *J–V* characteristics of hole‐only devices of commercial polymers and antimony(V) corrole, **2‐SCN**, at 300 K (*J* is scaled with sample thickness (*d*) and dielectric constant ($\varepsilon_{r}$)). c) Temperature‐dependent *J–V* characteristics of hole‐only devices of antimony(V) corrole, **2‐SCN** in the dark. Inset: Corresponding mobility (*μ*) versus 1/T characteristics. d) Photoresponse of a hole‐only device of antimony(V) corrole, **2‐SCN**. Inset: White light photoresponsivity values of different devices (the dotted line represents the average value). e) *J–V* characteristics of the single‐component PV cells under 1 Sun‐illumination. f) Comparison of open‐circuit voltage (*V*_oc_) of different OSCs.

The *J–V* characteristics of the hole‐only devices for antimony corrole(III), **1**, and antimony corrole(V), **2**, with different *β*‐substituents (SCN or H) at 300 K, are shown in **Figure** **5**a. A clear transition from the Ohmic to the SCLC regime is observed, with the trap density estimated to be ≈10^16^ cm^−3^. For both corroles, substituting SCN in place of H leads to an increase in the *μ*‐value. In the case of antimony corrole(III), **1**, the *μ*‐value shows nearly a twofold improvement, increasing from (1.70 ± 0.51) × 10^−4^ to (3.2 ± 2.2) × 10^−4^ cm^2^ V^−1^ s^−1^.

In contrast, approximately a two‐order‐of‐magnitude enhancement in charge carrier mobility was observed for antimony corrole (V), **2**, increasing from (5.36 ± 0.2) × 10^−6^ to (3.23 ± 0.82) × 10^−4^ cm^2^ V^−1^ s^−1^ This significant improvement highlights that SCN substitution effectively enhances the dielectric constant in antimony corroles while maintaining high bulk charge carrier mobility. To further confirm the donor nature of the corroles, electron‐only devices (ITO/antimony corroles/Ag) were fabricated using two representative molecules: antimony(III) corrole, **1‐H**, and antimony(V) corrole, **2‐SCN**, as described earlier. The corresponding characteristics of these electron‐only devices are shown in Figure S28, Supporting Information. The average electron mobilities of antimony(III) corrole, **1‐H**, and antimony(V) corrole, **2‐SCN**, at 300 K were determined to be (1.02 ± 0.15) × 10^−8^ and (2.62 ± 1.13) × 10^−7^ cm^2^ V^−1^ s^−1^, respectively.

These results suggest that both antimony corroles are more efficient as hole‐transporting layers (donor molecules) than as electron‐transporting layers (acceptor molecules). The highest mobility observed for antimony(V) corrole, **2‐SCN**, can be attributed to the high crystallinity of the molecule, as indicated by the XRD measurement (Figure S29, Supporting Information), and its planar structure (*D*_oop_ ≈ 0.3710 Å). Consistent with the structural ordering of antimony(V) corrole, **2‐SCN**, we observe an increase in the PLQY and fluorescence lifetime, indicating a reduction in nonradiative pathways and trap density. Furthermore, the temperature‐dependent *J–V* measurements of antimony(V) corrole, **2‐SCN**, devices exhibit a much lower $E_{a}$‐value of ≈94 meV, compared to antimony(III) corrole, **1‐H**, suggesting a lower energy barrier for the hopping of charge carriers (Figure 5c, S30, and S31, Supporting Information). To gain a comprehensive understanding of charge transport, we compared the hole mobilities of the corroles with those of commercially available polymers such as P3HT, PBTTT‐C14, and PCPDTBT. Devices fabricated from P3HT and PBTTT‐C14 donor polymers exhibit a mobility one order of magnitude lower (≈10^−5^ cm^2^ V^−1^ s^−1^), while PCPDTBT, a D–A‐type polymer, demonstrates bulk hole mobility (≈10^−4^ cm^2^ V^−1^ s^−1^) comparable to that of the corroles (Figure 5b and Table S7, Supporting Information).

Next, we utilize our best‐performing molecule, antimony(V) corrole, **2‐SCN**, which features broad absorption in the visible range, a high $\varepsilon_{r}$ value, and a high *μ* value to design a single‐component photodetector device. The device exhibits an outstanding photoresponse to white light illumination (0.1 Sun), with an average responsivity (averaged over seven devices) of ≈5.7 A W^−1^. Champion devices achieve values up to 10 A W^−1^, which is among the best for organic semiconducting molecules (Figure 5d). Under similar conditions, photodetectors fabricated with P3HT and PBTTT exhibited photoresponsivities of 191 and 65 mA W^−1^, respectively, whereas PCPDTBT did not exhibit any photoresponse under white light illumination (Table S7, Supporting Information), likely due to its band energetics. The exceptional photoresponse of antimony(V) corrole, **2‐SCN** is attributed to the combination of its large absorbance coefficients in the visible region, long‐lived excited states (both singlet and triplet), high hole mobility, and high $\varepsilon_{r}$ value, which together facilitate efficient dissociation of the photogenerated charge carriers.^[^ ^32^, ^33^, ^34^, ^35^ ^]^

### 2.7 PV Performance

We then fabricated a single‐component solar cell as a proof‐of‐concept device, to elucidate the role of SCN and antimony's oxidation state in PV performance. A single‐component solar cell was fabricated to ensure that the impact of the molecule's chemical design and its associated properties could be directly observed in the PV performance. We fabricated solar cell devices using all the corrole molecules (see Supporting Information for details on fabrication and measurements), with the *J–V* curves presented in Figure 5e and S33, Supporting Information. Devices based on antimony corrole(III), **1‐SCN**, and antimony corrole(V), **2‐SCN**, consistently demonstrated a *V*_oc_ of ≈0.7 V (Figure 5f), whereas devices using antimony corrole(III), **1‐H**, and antimony corrole(V), **2‐H** showed no significant *V*_oc_ (Figure S33, Supporting Information). This observation indicates that SCN substitution plays a major role in providing a high *V*_oc_ value. We further compared the device performance of antimony(III) corrole, **1‐SCN**, and antimony(V) corrole, **2‐SCN**, with single‐component devices fabricated from commercial semiconducting molecules such as P3HT and PC_61_BM (Figure 5e). Interestingly, the open‐circuit voltage of SCN‐substituted corroles reach values up to 0.72 V, which is at least three times higher than that of commercial P3HT/PC_61_BM‐based single component photovoltaic devices (Figure 5f and Table S8, Supporting Information). Note that the *J–V* characteristics exhibit a low fill factor, indicating the need for further optimization of the metal–semiconductor interface (Table S8, Supporting Information). The high open‐circuit voltage suggests effective splitting of the electron–hole pairs and the corresponding quasi‐Fermi levels.^[^ ^36^ ^]^ Incident photon‐to‐current conversion efficiency (IPCE) measurements were conducted to correlate the cell's discrete efficiency as a function of wavelength, closely aligning with the absorption spectra (Figure S32, Supporting Information). Inspired by the exceptional *V*_oc_ of antimony corrole(V), **2‐SCN**‐based single‐component devices, we also fabricated bilayer devices. These included both normal architecture (ITO/PEDOT: PSS/antimony(V)corrole, **2‐SCN/**PCBM/Al) and inverted architecture (ITO/ZnO/PCBM/ antimony(V) corrole, **2‐SCN**/ PEDOT: PSS/Au) using PC_61_BM (see experimental details in the Supporting Information). However, these devices did not exhibit any significant *V*_oc_ (Figure S34, Supporting Information), attributed to the absence of an energy offset in the band structure between antimony corrole(V), **2‐SCN**, and PC_61_BM (Figure S35, Supporting Information). Therefore, a completely new acceptor molecule needs to be designed to achieve a successful bilayer PV device. Overall, we present a molecular design guideline for developing high‐dielectric‐constant organic molecules, enabling enhanced photovoltage and white‐light responsivity.

## 3 Conclusion

The synthesis, structural characterization, and optoelectronic properties of four antimony corrole complexes, featuring two different oxidation states of antimony and two different *β*‐substituents, were reported to investigate the role of molecular engineering in enhancing optoelectronic performance. Interestingly, we observe that the incorporation of strong electron‐withdrawing SCN groups on the bipyrrole unit of the corrole increases the $\varepsilon_{r}$‐value, a result that is consistently verified through the charge density distribution over the molecule obtained from DFT calculations. Surprisingly, despite the enhanced dielectric constant, it was possible to retain excellent bulk hole mobility, which is higher than that of a range of commercial donor molecules, indicating the effectiveness of our molecular design. Moreover, the structurally engineered antimony(V) corrole, **2‐SCN**, exhibits champion optoelectronic properties, such as a high $\varepsilon_{r}$ value, high charge carrier mobility, and broad absorption in the visible range, leading to an outstanding photoresponse with an average white light responsivity of 10 A W^−1^, which is among the best for organic semiconducting molecules. This value significantly outperforms commercial silicon photodetectors and organic donor polymers, which typically exhibit an average photoresponsivity of 100–500 mA W^−1^. Single‐component solar cells fabricated from antimony(V) corrole, **2‐SCN**, with optimized properties, demonstrate an open‐circuit voltage that is three times greater than that of the commercial P3HT polymer. Overall, we propose that systematic molecular engineering, which involves substituting suitable functional groups to induce a dipole moment and modulating oxidation states, offers a promising strategy for designing molecules tailored for high‐performance optoelectronic applications.

## 4 Experimental Section

4.1

4.1.1

##### Materials

The precursor's pyrrole, *p*‐chloranil, 4‐formyl benzonitrile, and 4‐bromobenzaldehyde were purchased from Aldrich, USA. SbCl_3_ and pyridine were purchased from Merck, India. Other chemicals were of reagent grade. Hexane and CH_2_Cl_2_ were distilled from KOH and CaH_2_, respectively. High‐performance liquid chromatography‐grade solvents were used for spectroscopic and electrochemical studies. The free base corroles, 10‐(4‐bromophenyl)‐5,15‐bis(4‐cyanophenyl) corrole and {2,3,17,18‐*tetra*(thiocyano)‐10‐(4‐bromophenyl)‐5,15‐bis(4‐cyanophenyl) corrole, were prepared according to the published procedures.^[^ ^22^, ^37^ ^]^ 2,3,17,18‐Tetrathiocyano‐10‐(4‐bromophenyl)‐5, 15‐bis(4‐cyanophenyl)corrolato‐antimony(III), (Antimony(III) corrole, **1‐SCN)** was prepared according to the published procedures.^[^ ^20^ ^]^ {10‐(4‐bromophenyl)‐5,15‐bis(4‐cyanophenyl) corrolato} (*trans‐*difluoro) antimony(V), (Antimony(V) corrole, **2‐H)** was prepared by following a methodology developed earlier by Gross et al.^[23h]^

##### Physical Measurements

UV‐vis spectral studies were performed on a Perkin−Elmer LAMBDA‐750 spectrophotometer. The elemental analyses were carried out with a Euro EA elemental analyzer. Emission spectra were measured on an Edinburgh FLS920 spectrofluorometer equipped with a photomultiplier tube (PMT) 980 for the visible and a Ge detector for emission in the NIR spectral region, using a 1 cm path length optical cell. Emission quantum yields were measured following Demas and Crosby method (the standard used: coumarin 153 in ethanol, Φ_fl_ = 0.55; and tetraphenyl‐porphyrin in toluene, Φ_fl_ = 0.11; respectively).^[^ ^38^, ^39^ ^]^ Emission spectra at the NIR region were recorded on HORIBA's new Fluorolog‐QM spectrofluorometer with an LN_2_‐cooled NIR PMT detector. The singlet‐oxygen quantum yield (^1^O_2_) was measured using a carbon tetrachloride solution of TPP as the reference with singlet‐oxygen production yield of 62% {Φ(^1^O_2_)_TPP_ = 0.62 in CCl_4_}. The singlet‐oxygen emissions were detected and quantified in the 1220–1330 nm range.^[^ ^40^ ^]^ The FLIM study used a time‐resolved confocal microscope (MicroTime 200, PicoQuant) using thin‐film blends of PVDF and antimony(III) corrole, **1‐H** or antimony(V) corrole, **2‐SCN** (details in Appendix 1 and 2, Supporting Information). The excitation source was a pulsed diode laser (*λ*_ex_ = 422 nm). FT−IR spectra were recorded on a Perkin−Elmer spectrophotometer with samples prepared as KBr pellets. The NMR measurements were carried out using a Bruker AVANCE 400 NMR spectrometer. With tetramethylsilane (TMS) as the internal standard, electrospray mass spectra were recorded on a Bruker Micro TOF‐QII mass spectrometer. Cyclic voltammetry was measured using a CS350 electrochemical test system (China). A glassy carbon working electrode, a platinum wire as an auxiliary electrode, and an Ag–AgCl reference electrode were used in a three‐electrode configuration. Tetrabutylammonium perchlorate (TBAP) was the supporting electrolyte (0.1 M), and the solution concentration was 10^−3^ M for the complex. The half‐wave potential $E_{298K}^{0}$ was set equal to 0.5 (*E*_pa_ + *E*_pc_), where *E*_pa_ and *E*_pc_ were anodic and cathodic cyclic voltammetric peak potentials, respectively. The scan rate was set as 100 mV s^−1^.

##### Crystal Structure Determination

Single crystals of antimony(V) corrole, **2‐SCN**, were grown by slow diffusion of antimony(V) corrole, **2‐SCN**, in CH_2_Cl_2_ solution into hexane, followed by slow evaporation under atmospheric conditions. The crystal data of antimony(V) corrole, **2‐SCN**, were collected on a Rigaku Oxford diffractometer at 100 K. Selected data collection parameters and other crystallographic results are summarized in Table S1, Supporting Information. All data were corrected for Lorentz polarization and absorption effects. The program package SHELXTL^[^ ^41^ ^]^ was used for structure solution and full‐matrix least‐squares refinement on F^2^. Hydrogen atoms were included in the refinement using the riding model. Contributions of H atoms for the water molecules were included but were not fixed. Disordered solvent molecules were taken out using the SQUEEZE command in PLATON.^[^ ^42^ ^]^ CCDC 2288716 contains the supplementary crystallographic data for antimony(V) corrole, **2‐SCN**. These data can be obtained free of charge via www.ccdc.cam.ac.UK/data_request/cif.

##### Experimental Section

Refer to the Supporting Information.

##### Device Fabrication

Details are provided in the Supporting Information.

##### Synthesis: *Synthesis of {10*‐*(4*‐*bromophenyl)*‐*5,15*‐*bis(4*‐*cyanophenyl) Corrolato}antimony(III),* (Antimony(III) Corrole, **1‐H)**

Antimony(III) corrole, **1‐H**, was prepared by following a general synthetic protocol for the synthesis of antimony(III)‐corrole complexes. 100 mg (0.152 mmol) of 10‐(4‐bromophenyl)‐5,15‐bis(4‐cyanophenyl)corrole was dissolved in 25 mL of pyridine. After that, 250 mg (1.09 mmol) of SbCl_3_ was added to the solution, and the reaction mixture was heated to reflux at 120 °C. The reaction continued until the fluorescence of the starting corrole had not fully disappeared. A rotary evaporator dried the solvent, and the crude reaction mixture was purified by silica gel (100–200 mesh) column chromatography {2% CH_3_CN + 98% CH_2_Cl_2_ mixture as eluent). The final compound was recrystallized by CH_2_Cl_2_ and hexane mixture and was collected as pure crystalline antimony(III) corrole, **1‐H**.

Yield 94 mg (0.121 mmol, 80%). Anal. Calcd (found) for C_39_H_20_BrN_6_Sb (**1**): C, 60.50 (60.37); H, 2.60 (2.71); N, 10.85 (10.77). UV/Vis spectra (CH_2_Cl_2_) λ_max_/nm (*ε*/*M* ^−1^ cm^−1^): 446(129014), 462(100852), 542(11293), 575(9157), 612(12082), 664(35253) (Figure 2). ^1^H NMR (700 MHz, Chloroform‐*d*) *δ* 9.26 (d, *J* = 4.0 Hz, 2H), 9.05 (d, *J* = 4.6 Hz, 2H), 8.78 (m, 4H), 8.36 (s, 4H), 8.08 (d, *J* = 7.6 Hz, 5H), 7.89 (d, *J* = 50.9 Hz, 3H) (Figure S3, Supporting Information). ^13^C {^1^H} NMR (176 MHz, Chloroform‐*d*) *δ* 145.36, 143.23, 140.29, 138.77, 137.60, 136.31, 135.29, 135.04, 131.55, 130.80, 128.43, 125.79, 125.45, 122.42, 119.24, 117.17, 115.17, 111.70, 109.50 (Figure S4, Supporting Information). ESI‐HRMS (in acetonitrile): *m/z* = 771.9943 for [**M**]^**+**^ (771.9971 calcd for C_39_H_20_BrN_6_Sb) (Figure S8, Supporting Information).

##### Synthesis: Synthesis of {2,3,17,18‐Tetra(thiocyano)‐10‐(4‐bromophenyl)‐5,15‐bis(4‐cyanophenyl) Corrolato} (Trans‐Difluoro) Antimony(V), (Antimony(V) Corrole, **2‐SCN**)

30 mg (0.033 mmol) of 2,3,17,18‐tetrathiocyano‐10‐(4‐bromophenyl)‐5,15‐bis(4‐cyanophenyl)corrole was dissolved in 25 mL of pyridine and the solution was stirred for 2 min. 70 mg (0.30 mmol) of *antimony trichloride* was added to the stirred solution, and then the reaction mixture was heated to reflux for half an hour. The solvent was evaporated to dryness using a rotary evaporator. The crude reaction mixture was then dissolved in 20 mL of CH_2_Cl_2_—CH_3_CN (10:1) mixture, and the excess of NOBF_4_ (80 mg, 0.68 mmol) was added to the reaction mixture. The color of the reaction mixture changed immediately from yellowish‐green to bluish‐green. Then, the crude reaction mixture was immediately purified through a previously packed silica gel column (95% CH_2_Cl_2_ and 5% CH_3_CN mixture as eluent). The pure fraction was recrystallized from the CH_2_Cl_2_/hexane mixture to give 19 mg (0.018 mmol) of pure antimony(V) corrole, **2‐SCN**.

Yield 19 mg (0.018 mmol, 55%). Anal. Calcd (found) for C_43_H_16_BrN_10_F_2_S_4_Sb (**1**): C, 49.63 (49.74); H, 1.55 (1.70); N, 13.46 (13.61). UV/Vis spectra (CH_2_Cl_2_) λ_max_/nm (*ε*/*M* ^−1^ cm^−1^): 410(101822), 435(104925), 531(8986), 552(14459), 574(30832), 595(37414), 631(78875) (Figure 2). ^1^H NMR (400 MHz, Chloroform‐*d*) *δ* 8.84–8.71 (m, 4H), 8.29 (d, *J* = 7.9 Hz, 4H), 8.19 (d, *J* = 7.8 Hz, 4H), 7.97 (q, *J* = 8.4 Hz, 4H) (Figure S5, Supporting Information). ^13^C {^1^H} NMR (176 MHz, Chloroform‐*d*) *δ* 147.94, 141.05, 140.11, 135.50, 135.36, 134.69, 134.03, 132.81, 132.31, 132.14, 130.37, 128.00, 124.83, 123.91, 118.83, 118.40, 118.15, 115.02, 112.98, 108.85, 108.38. (Figure S6, Supporting Information). ^19^F {^1^H} NMR (377 MHz, Chloroform‐*d*) *δ* − 98.61 (s, 2 F) (Figure S7, Supporting Information). ESI‐HRMS (in acetonitrile): *m/z* = 1060.8589 for [**M** + Na]^+^ (1060.8524 calcd for C_43_H_16_BrN_10_F_2_S_4_SbNa) (Figure S9, Supporting Information). Antimony(V) corrole, **2‐SCN** displayed strong fluorescence at 654 nm in CH_2_Cl_2_ (Figure 2).

##### Synthesis: Synthesis of {10‐(4‐bromophenyl)‐5,15‐bis(4‐cyanophenyl) Corrolato} (Trans‐Difluoro) Antimony(V), (Antimony(V) Corrole, 2‐H)

To synthesize compound antimony(V) corrole, **2‐H**, 20 mg of {10‐(4‐bromophenyl)‐5,15‐bis(4‐cyanophenyl) corrolato}antimony(III), (Antimony(III) corrole, **1‐H**), was reacted with aqueous HF (12 mL) in a 25 mL CH_2_Cl_2_ solution. The mixture was stirred at room temperature for 24 h. Once the starting materials were completely consumed, the reaction was halted, and the solution was filtered through a prepacked silica gel column using CH_2_Cl_2_ as the eluent. Further, antimony(V) corrole, **2‐H** purification was achieved through recrystallization from a CH_2_Cl_2_/hexane mixture.

Yield 15 mg (0.018 mmol, 69%). UV/Vis spectra (CH_2_Cl_2_) λ_max_/nm (*ε*/*M* ^−1^ cm^−1^): 418 (97 289), 532 (4420), 568 (6130), 606 (16 929) (Figure S15, Supporting Information). ^1^H NMR (400 MHz, Chloroform‐*d*) *δ* 9.65 (d, *J* = 4.2 Hz, 2H), 9.26 (d, *J* = 4.7 Hz, 2H), 9.17 (d, *J* = 4.2Hz, 2H), 9.11 (d, *J* = 4.7Hz, 2H), 8.52 (d, *J* = 8.0 Hz, 4H), 8.18 (t, *J* = 8.1 Hz, 6H), 8.00 (d, *J* = 8.1 Hz, 2H) (Figure S16a, Supporting Information). ^13^C NMR (101 MHz, CDCl_3_) *δ* 143.97, 140.55, 138.50, 137.01, 136.09, 135.45, 134.51, 134.31, 132.02, 131.36, 130.40, 129.64, 128.94, 127.37, 123.53, 120.47, 118.91, 115.28, 112.74, 110.49 (Figure S16b, Supporting Information). ^19^F NMR (377 MHz, CDCl_3_) *δ*‐104.86 (Figure S16c, Supporting Information). ESI‐HRMS (in acetonitrile): *m/z* = 809.9742 for [**M**]^+^ (809.9939 calcd for C_39_H_20_BrN_6_F_2_Sb) (Figure S17, Supporting Information).

## Conflict of Interest

The authors declare no conflict of interest.

## Author Contributions

**Tanmoy Pain**: data curation: (lead); formal analysis: (lead); writing—original draft: (lead). **Md. Saifuddin**: data curation: (lead); formal analysis: (lead); writing—original draft: (lead). **Anshuman Sahoo**: data curation: (supporting); formal analysis: (supporting). **Biplab Mahapatra**: ; data curation: (supporting); formal analysis: (supporting). **Subhajit Kar**: data curation: (supporting); formal analysis: (supporting). **Rwiddhi Chakraborty**: data curation: (supporting); formal analysis: (supporting). **Satyaprasad P. Senanayak**: conceptualization: (lead); writing—original draft: (lead); writing—review & editing: (lead). **Sanjib Kar**: conceptualization: (lead); writing—original draft: (lead); writing—review & editing: (lead).

## Supporting information

Supplementary Material

## Data Availability

The data that support the findings of this study are available in the supporting information of this article.
