# Supplementary material for: Molecular Engineering for Enhancing the Dielectric and Optoelectronic Properties of Antimony Corroles
Source: Small Sci. 2025 Mar 4;5(6):2400589. doi: 10.1002/smsc.202400589 (PMC12168605; doi:10.1002/smsc.202400589)
Supplement: Supplementary file 1 — Supplementary Material [file SMSC-5-2400589-s001.pdf]

Supporting Information

Molecular Engineering for Enhancing the Dielectric and Opto-electronic Properties of Antimony Corroles

Tanmoy Pain,[1,2] Md Saifuddin,[2,3] Anshuman Sahoo,[2,3] Biplab Mahapatra,[2,3] Subhajit Kar,[1,2] Rwiddhi Chakraborty,[1,2] Satyaprasad P Senanayak,[* 2,3] and Sanjib Kar[* 1,2]

[1]*School of Chemical Sciences, National Institute of Science Education and Research (NISER), Bhubaneswar – 752050, India, An OCC of HBNI, Jatni 752050, India.*
E-mail: sanjib@niser.ac.in

[2]*Centre for Interdisciplinary Sciences, National Institute of Science Education and Research Bhubaneswar, Jatni, Khurda, Odisha 752050, India*

[3]*Nanoelectronics and Device Physics Lab, School of Physical Sciences, National Institute of Science Education and Research, An OCC of HBNI, Jatni 752050, India.*
E-mail: satyaprasad@niser.ac.in

EXPERIMENTAL SECTION

Computational Methods

All calculations were performed by using the Gaussian 06 software. [1] The geometry optimization of antimony corroles (antimony (III) corrole, **1-H** and antimony(V) corrole, **2-SCN**) was performed at the B3LYP/6-311G(d,p) level of theory. The LANL2DZ pseudopotential was used for the Sb atom. The TD-DFT calculations were performed at the B3LYP level of theory, and 6-G11 (d, p) basis sets using Gaussian-06 software. A PCM solvent model was used to calculate DCM as the solvent.

Device Fabrication

For hole-only devices (ITO/PEDOT: PSS/ antimony corroles /Au), thin films of all the corroles were coated on pre-cleaned ITO glass substrates mainly using drop-cast or spin coating methods. Initially, The PEDOT: PSS layer (from Ossila) was introduced on ITO-coated glass by spin coating at 2000 rpm for 60 seconds in ambient conditions and annealed at 110 °C for 5 minutes. Antimony (III) corrole, **1-H** films were prepared by dissolving it in chlorobenzene (CB) with a concentration of 10 mg/mL and was drop casted on the PEDOT: PSS coated ITO substrates after which the films were annealed at 110 °C for 20 minutes followed by 60°C for 1 hour. Thin films of antimony(III) corrole, **1-SCN** (20 mg/mL in CB) and antimony(V) corrole, **2-H** (20 mg/mL in CB) were coated on the PEDOT: PSS coated ITO glass substrates using drop-cast technique. Both the films were annealed at 80°C for 1 hour. However, antimony(V) corrole, **2-SCN** (20 mg/mL in CB) films were obtained by spin coating at1500 rpm for 60 s and the films were annealed at 100 °C for 20 minutes. The devices were then completed by evaporating Au electrodes of 25 nm thickness using a thermal evaporation technique (deposition rate ~ 0.3 Å/s and chamber pressure ~10^{-6} mbar). For electron-only devices (ITO/antimony corrole/Ag), thin films of antimony(III) corrole, **1-H** and antimony(V) corrole, **2-SCN** were introduced on ITO substrates using the same procedure as described earlier followed by thermal deposition of Ag electrode of 25 nm thickness using a thermal evaporation technique (deposition rate ~ 0.3 Å/s and chamber pressure ~10^{-6} mbar). Hole-only devices of other donor polymers such as Poly(3-hexylthiophene) (P3HT), Poly[2,5-bis(3-tetradecylthiophen-2-yl)thieno[3,2-b]thiophene] (PBTTT-C14) and Poly[2,6-(4,4-bis-(2-ethylhexyl)-4H-cyclopenta[2,1-b;3,4-b′]dithiophene)-alt-4,7(2,1,3-benzothiadiazole)] (PCPDTBT) were fabricated using the same device structure as utilized for the corrole molecules . Solutions of P3HT and PCPDTBT of concentration 4 mg/mL were made using CB as a solvent, whereas PBTTT-C14 solution of similar

concentration was made using 1,2-Dichlorobenzene. Thin films of all the polymers were formed by drop casting method and films were annealed at 80 °C for 3 hrs.

I-V Measurements: All I-V measurements were carried out under vacuum (10^{-5} - 10^{-6} mbar) in a Lake Shore probe station utilizing a Keysight B1500A semiconductor parameter analyzer. Temperature-dependent measurements were obtained over a range of 300 K to 100 K in steps of 10 K using the closed cycle helium cryostat of the Lakeshore probe station. To check the photoresponse of antimony corroles and the polymers, we illuminated the devices with white light of 0.1 Sun (10 mW/cm^2) intensity. The photoresponsivity is defined as : $\frac{I_{illumination} - I_{dark}}{Power}$ where the current is obtained at +10V.

Dielectric constant measurement: The capacitance module of the B1500A semiconductor parameter analyzer was utilized to obtain the *C*-*f* data of the devices over a frequency range of 1 kHz to 5 MHz. For the measurement, the DC bias was kept at 0 V, while the AC bias was kept at 100 mV.

Thickness Measurement: The thicknesses of the corrole thin films were measured using a Bruker DektakXT step profiler.

Single Component solar cell fabrication:

PEDOT:PSS hole transport layer (HTL) was spin coated at 4000 rpm for 30s on precleaned patterned ITO substrates. The films were then annealed at 120°C for 20 minutes and then taken immediately into the glovebox for subsequent deposition of organic thin films Antimony(III) corrole, **1-H** (concentration = 20 mg/ml), antimony(III) corrole, **1-SCN** (concentration = 20 mg/ml), and antimony(V) corrole, **2-H** (concentration = 10 mg/ml) was drop casted using CB as a solvent. For P3HT, 4 mg/ml solution in CB was spin coated at 500 rpm for initial 30s and at 1500 rpm for next 30s two times. Antimony(V) corrole, **2-SCN** (concentration = 20 mg/ml) and PCBM (concentration = 10 mg/ml) was drop casted using CB as a solvent. All the films were annealed at 80°C for 60 minutes. For the electron transport layer ZnO was prepared by dissolving 314 mg of zinc acetate dihydrate [$Zn(CH_3COO)_2.2H_2O$] in 3.14 ml of 2-methoxyethanol and 86 µl of ethanolamine as stabilizer. The solution was stirred for 1 h at 65°C and left for 3 h to form a gel. This solution was spin coated at 2000 rpm for 60s on the active layer followed by of annealing at 120°C for 2 hrs. 100 nm Al electrode were deposited by thermal evaporation to complete the single component solar cell structure.

Bilayer Solar Cell Fabrication:

Normal bilayer structure: PEDOT:PSS as hole transport layer (HTL) was spin coated at 4000 rpm for 30s and annealed at 120°C for 20 min. Antimony(V) corrole, **2-SCN** solution of concentration 10 mg/mL in CB were drop casted and the films were then annealed at 80°C for 1 hr. Then, PCBM (10 mg/mL in CB) was spin coated at 1000 rpm for 30s and the films were annealed at 80°C for 30 minutes. Due to the non-availability of orthogonal solvents for these bi-layer structures, we fabricated a thicker layer of Antimony(V) corrole, **2-SCN**, such that PCBM coating from chlorobenzene would not completely remove the donor layer. 100 nm Al electrodes were deposited by thermal evaporation to complete the solar cell bilayer structure.

Inverted bilayer structure: ZnO as electron transport layer (ETL) was spin-coated at 2000 rpm for the 60s on precleaned patterned ITO substrates. The films were then annealed at 120°C for 120 minutes. PCBM (10 mg/mL in CB) was spin-coated at 1000 rpm for 60 s, and films were annealed at 80°C for 30 minutes. After that, antimony(V) corrole, **2-SCN** solution of concentration 20 mg/mL in a mixed solvent (90% methanol + 10% DCM) was spin-coated at 1500 rpm for the 60s. Then the films were annealed at 80°C for 30 minutes. Further, PEDOT: PSS as hole transport layer (HTL) was spin-coated at 4000 rpm for 30 s and annealed for 20 min at 120°C. 50 nm Au electrodes were deposited by thermal evaporation to complete the inverted solar cell bilayer structure.

Solar cell measurements: A solar simulator (SciSun-300) was used to illuminate the solar cells (active area ~ 3 mm^2) at an intensity of 100 mW/cm^2, and the I-V characteristics were recorded using a Keithley 2450 source meter.

Error Analysis: In general, typical device responses such as bulk charge carrier mobility, dielectric constant, and photo response measurements are performed on 8 – 10 devices, and the mean value is reported. The error bars are representative of the standard deviation in the measurement.

References:

[1] M. J. Frisch, G. W. Trucks, H. B. Schlegel, G. E. Scuseria, M. A. Robb, J. R. Cheeseman, G. Scalmani, V. Barone, G. A. Petersson, H. Nakatsuji, X. Li, M. Caricato, A. Marenich, J. Bloino, B. G. Janesko, R. Gomperts, B. Mennucci, H. P. Hratchian, J. V. Ortiz, A. F. Izmaylov, J. L. Sonnenberg, D. Williams-Young, F. Ding, F. Lipparini, F. Egidi, J. Goings, B. Peng, A. Petrone, T. Henderson, D. Ranasinghe, V. G. Zakrzewski, J. Gao, N. Rega, G. Zheng, W. Liang, M. Hada, M. Ehara, K. Toyota, R. Fukuda, J. Hasegawa, M. Ishida, T. Nakajima, Y. Honda, O. Kitao, H. Nakai, T. Vreven, K. Throssell, J. A. Montgomery, Jr., J. E. Peralta, F. Ogliaro, M. Bearpark, J. J. Heyd, E. Brothers, K. N. Kudin, V. N. Staroverov, T. Keith, R. Kobayashi, J. Normand, K. Raghavachari, A. Rendell, J. C. Burant, S. S. Iyengar, J. Tomasi, M. Cossi, J. M. Millam, M. Klene, C. Adamo, R. Cammi, J. W. Ochterski, R. L. Martin, K. Morokuma, O. Farkas, J. B. Foresman, and D. J. Fox, Gaussian, Inc., Wallingford CT, **2016**.

Contents:

**Table S1**	Crystallographic Data for antimony(V) corrole, **2-SCN**.
**Table S2**	UV–Vis. data for antimony corroles in dichloromethane.
**Table S3**	Contributions of various interactions in percentage to Hirshfeld surface area in antimony(V) corrole, **2-SCN.**
**Table S4**	TD-DFT Calculated Electronic Transitions for Antimony(III) corrole, **1-H**.
**Table S5**	TD-DFT Calculated Electronic Transitions for antimony(V) corrole, **2-SCN**.
**Table S6**	Calculated dipole (in Debye) and quadrupole moments (in Debye-Ang): The dipole and quadrupole moments (anisotropic values) are calculated by DFT, where Qπ is the out-of-plane component and is perpendicular to the molecular plane.
**Table S7**	Parameters obtained from hole-only devices of different p-type polymers and antimony corroles (Antimony(III) corrole, **1-H** and antimony(V) corrole, **2-SCN**). Average values are measured from 6 devices in each case.
**Table S8**	Device performance parameters of the single-component cells. Average parameters are obtained from 3 devices.
**Figure S1**	FT-IR spectrum of antimony(III) corrole, **1-H** as a KBr pellet.
**Figure S2**	FT-IR spectrum of antimony(V) corrole, **2-SCN** as a KBr pellet.
**Figure S3**	^{1}H NMR (700 MHz) spectrum of antimony(III) corrole, **1-H** in $CDCl_3$.
**Figure S4**	^{13}C NMR {^{1}H} (176 MHz) spectrum of antimony(III) corrole, **1-H** in $CDCl_3$.
**Figure S5**	^{1}H NMR (400 MHz) spectrum of antimony(V) corrole, **2-SCN** in $CDCl_3$.
**Figure S6**	^{13}C NMR {^{1}H} (176 MHz) spectrum of antimony(V) corrole, **2-SCN** in $CDCl_3$.
**Figure S7**	^{19}F NMR {^{1}H} (377 MHz) spectrum of antimony(V) corrole, **2-SCN** in $CDCl_3$.
**Figure S8**	ESI- MS spectrum of antimony(III) corrole, **1-H** in CH_3CN shows the measured spectrum with an isotopic distribution pattern.
**Figure S9**	ESI- MS spectrum of antimony(V) corrole, **2-SCN** in CH_3CN shows the measured spectrum with an isotopic distribution pattern.

Figure S10 Fingerprint plots of antimony(V) corrole, **2-SCN** with different interactions are highlighted in color.

Figure S11 (a) Hirshfeld surface, and (b) supramolecular assemblies in antimony(V) corrole, **2-SCN**.

Figure S12 (a) Hirshfeld surface, and (b) hydrogen bonding interactions in antimony(V) corrole, **2-SCN**.

Figure S13 Cyclic voltammograms of antimony(III) corrole, **1-H** in dichloromethane. The potentials are *vs.* Ag/AgCl.

Figure S14 Cyclic voltammograms and differential pulse voltammograms of antimony(V) corrole, **2-SCN** in dichloromethane. The potentials are vs. Ag/AgCl.

Figure S15 Electronic absorption spectrum of antimony(V) corrole, **2-H** in DCM.

Figure S16 a) ^{1}H NMR (400 MHz) spectrum of antimony(V) corrole, **2-H** in $CDCl_3$.

Figure S16 b) ^{13}C NMR {^{1}H} (176 MHz) spectrum of antimony(V) corrole, **2-H** in $CDCl_3$.

Figure S16 c) ^{19}F NMR {^{1}H} (377 MHz) spectrum of antimony(V) corrole, **2-H** in $CDCl_3$.

Figure S17 ESI- MS spectrum of antimony(V) corrole, **2-H** in CH_3CN shows the measured spectrum with an isotopic distribution pattern.

Figure S18 Electronic absorption spectrum of antimony corroles in solution and thin film.

Figure S19 DFT-optimized geometry of antimony(III) corrole, **1-H** using the 6-311G (d, p) basis set.

Figure S20 TD-DFT-based electronic absorption spectra of antimony(III) corrole, **1-H.**

Figure S21 DFT-optimized geometry of Antimony(V) corrole, **2-SCN** using the 6-311G (d, p) basis set.

Figure S22 TD-DFT-based electronic absorption spectra of antimony(V) corrole, **2-SCN**.

Appendix-1 FLIM and Dielectric properties measurements of Antimony corroles and PVDF-HFP mixed system.

Figure S23 Fluorescence lifetime images for blended films of PVDF: HFP with antimony(V) corrole, **2-SCN** in ratio (a) 1:1 and (b) 1:2.

Figure S24 Fluorescence lifetime image for (b) pristine films of antimony(III) corrole, **1-H**; blended films of PVDF: HFP with antimony(III) corrole, **1-H** in ratio (c) 1:1, (d) 1:2, (e) 1:3. (f) The plot of the fluorescence lifetime for different films is estimated to be 476 ps, 724 ps, and 642 ps, respectively.

Figure S25 Fluorescence lifetime distribution (fitted with Gaussian function) or pristine antimony (III) corrole, **1-H** blended with increasing PVDF-HFP amounts (1:1, 1:3, 1:5).

Figure S26 Fluorescence lifetime distribution (fitted with Gaussian function) for pristine antimony(V) corrole, **2-SCN** and blended with increasing PVDF-HFP amount with different ratios.

Appendix-2 Dielectric properties measurements of the thin films of silicon substrates by taking different volume ratios of both antimony corroles (Antimony(III) corrole, **1-H** and Antimony(V) corrole, **2-SCN**) and PVDF-HFP

Figure S27 Frequency-dependent dielectric constant plots of PVDF-HFP and a mixture of antimony(V) corrole, **2-SCN** and PVDF-HFP in 1:1 and 1:2 ratios.

Appendix-3 Charge transport measurement.

Figure S28 J-V characteristics of electron-only devices of antimony corroles (Antimony(III) corrole, **1-H** and antimony(V) corrole, **2-SCN**) (*J* is scaled with sample thickness (d) and dielectric constant (ε_r).

Appendix-4 XRD measurement

Figure S29 p-XRD pattern of antimony corroles (Antimony(III) corrole, **1-H** and Antimony(V) corrole, **2-SCN**).

Appendix-5 Temperature dependent charge transport measurements

Figure S30 Temperature-dependent J-V characteristics of hole-only devices of antimony(III) corrole, **1-H** under dark conditions. Inset: Corresponding μ versus 1/T characteristics.

Figure S31 J-V characteristics of hole-only devices of antimony(V) corrole, **2-SCN** under light illumination. Inset: Corresponding μ versus 1/T characteristics.

Appendix-6 Solar Cell Measurement of single layer and bilayer structure

Figure S32 The IPCE measurement of antimony(V) corrole, **2-SCN** obtain from the photovoltaic devices.

Figure S33 J–V characteristics of the single-component photovoltaic cell of antimony(III) corrole, **1-H and** antimony(V) corrole, **2-H** under 1 sun illuminated condition.

Figure S34 **(a)** J–V characteristics of the normal bilayer photovoltaic cell (multiple devices) of antimony(V) corrole, **2-SCN** under 1 sun illuminated condition. **(b)** J–V characteristics of the inverted bilayer photovoltaic cell (multiple devices) of antimony(V) corrole, **2-SCN**. All the curves are shown here were taken under 1 sun illuminated condition.

Figure S35 Energy level diagram of antimony(V) corrole, **2-SCN** as donor and $PC_{61}BM$ as acceptor.

Appendix-7 Optimized Cartesian Co-ordinates of antimony(III) corrole, **1-H**.

Appendix-8 Optimized Cartesian Co-ordinates of antimony(V) corrole, **2-SCN**.

Table S1 Crystallographic data for antimony(V) corrole, **2-SCN**.

Compound code	Antimony(V) corrole, **2-SCN**
molecular formula	C_{43} $H_{16}BrF_2N_{10}S_4Sb$, CH_2Cl_2
Fw	1125.48
Radiation	Mo *K*α
Crystal system	Monoclinic
space group	$P2_1/c$
a(Å)	11.8455(5)
b(Å)	14.0126(4)
c(Å)	28.2862(10)
a(deg)	90
β(deg)	98.636(4)
g(deg)	90
V(Å^3)	4641.9(3)
Z	4
μ (mm^{-1})	1.803
T(K)	100
D_{calcd} (g cm^{-3})	1.610
2θ range (deg)	6.504 to 53.462
e data (R_{int})	9781 (0.0761)
R1 (I>$2\sigma(I)$)	0.0910
WR2 (all data)	0.2514
GOF	1.027
$\Delta\rho_{max}$, $\Delta\rho_{min}$ (e Å^{-3})	3.52, -1.78

Table S2 UV−Vis. data for antimony corroles in dichloromethane.

Compound	UV−vis. Data [a] λ_{max} / nm (ε / 10^5 $M^{-1}cm^{-1}$)
Antimony(III) corrole, **1-H**	446 (129014), 462 (100852), 542 (11293), 575 (9157), 612 (12082), 664 (35253)
Antimony(III) corrole, **1-SCN**	425(46650), 447(56600), 474(111050), 551(17850), 592(28100), 655(25900), 714(77850)
Antimony(V) corrole, **2-H**	418 (97289), 532 (4420), 568 (6130), 606 (16929)
Antimony(V) corrole, **2-SCN**	410 (101822), 435 (104925), 531 (8986), 552 (14459), 574 (30832), 595 (37414), 631 (78875)

[a]In dichloromethane. [a] Error limits : λ_{max}, ± 1 nm and ε, ± 10%.

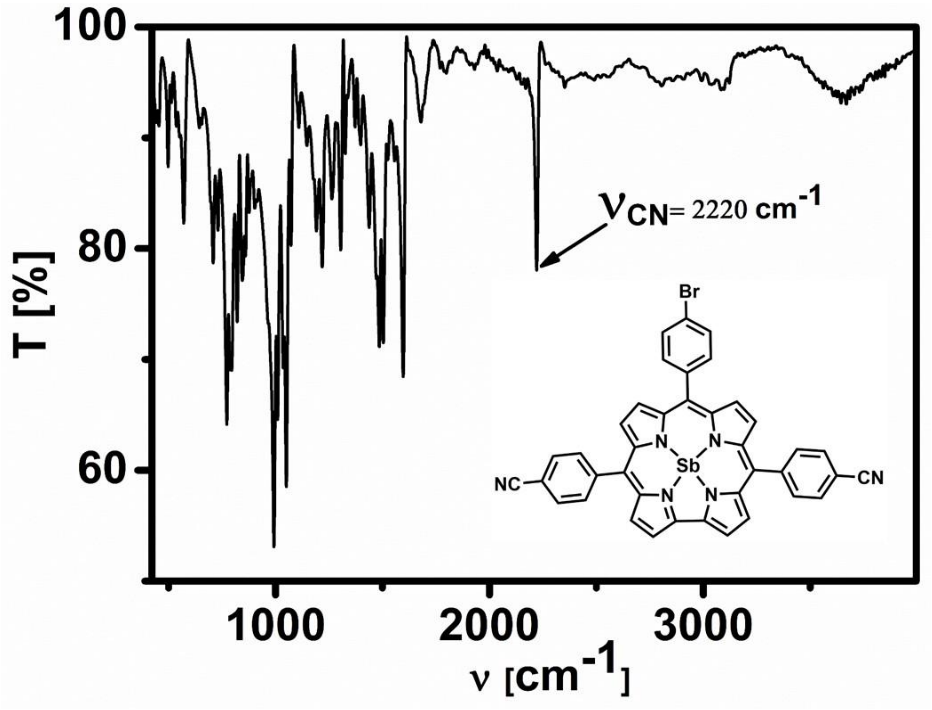

Figure S1 FT-IR spectrum of Antimony(III) corrole, **1-H** as a KBr pellet.

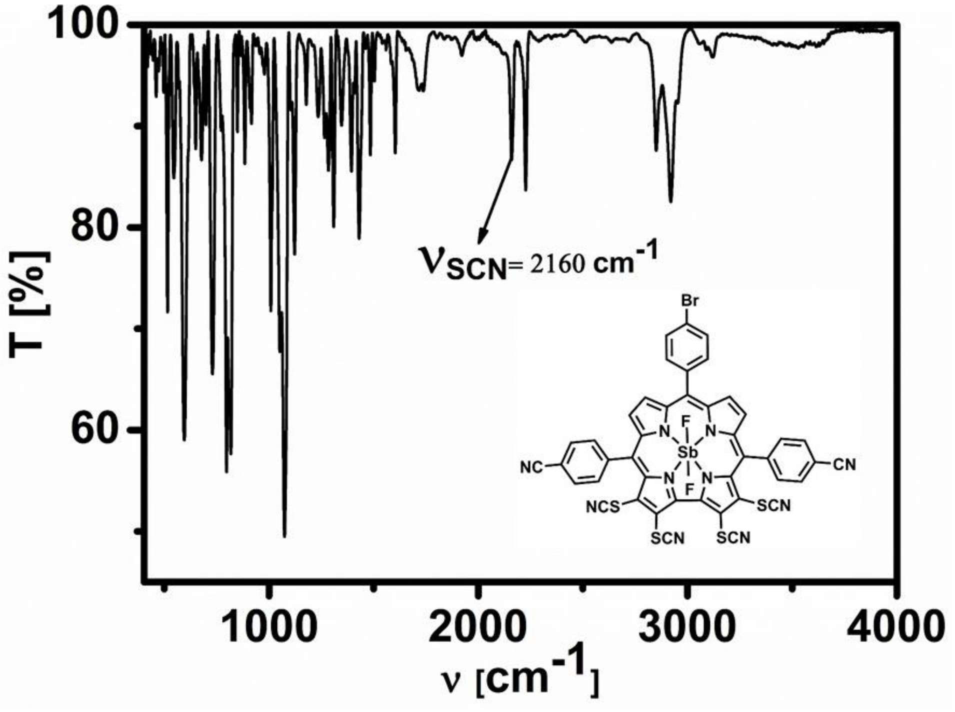

Figure S2 FT-IR spectrum of antimony(V) corrole, **2-SCN** as a KBr pellet.

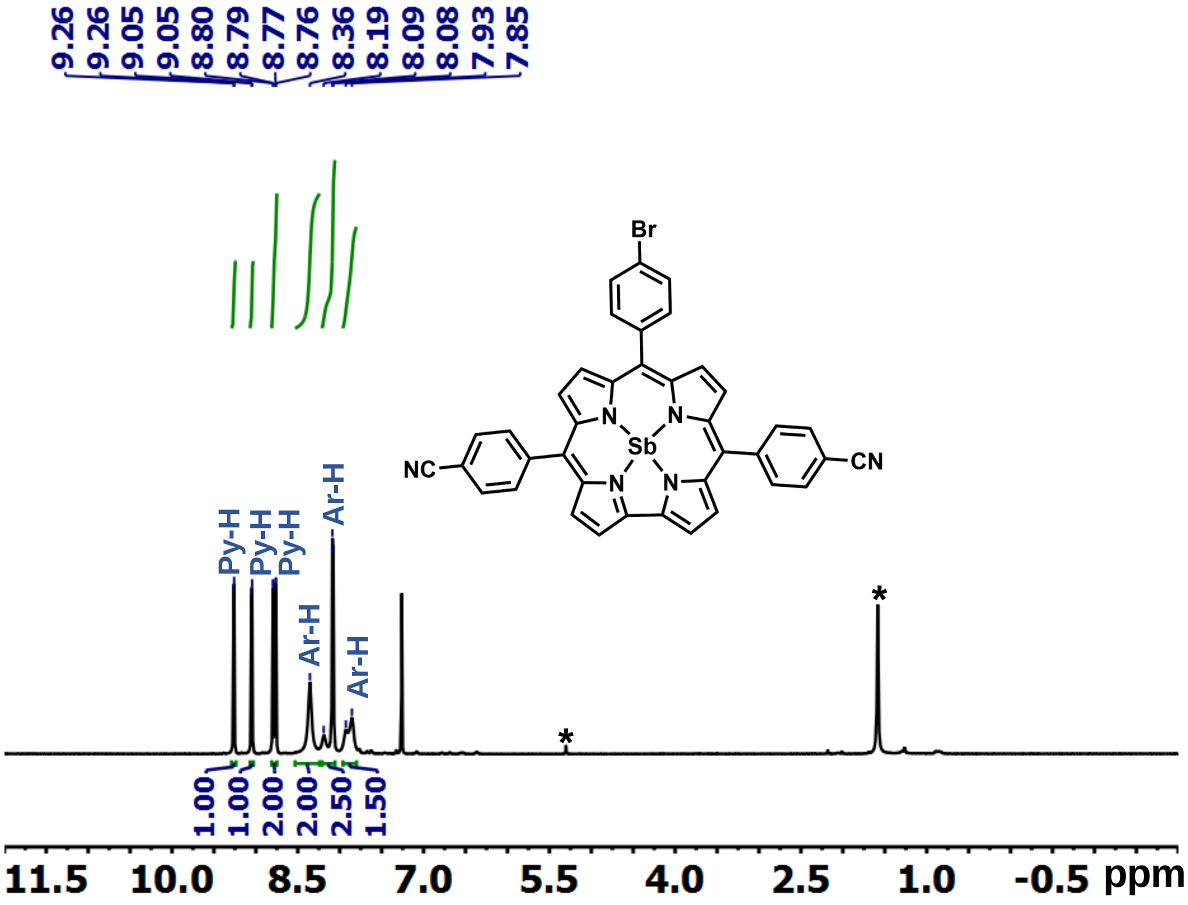

Figure S3 ^{1}H NMR (700 MHz) spectrum of antimony(III) corrole, **1-H** in $CDCl_3$.

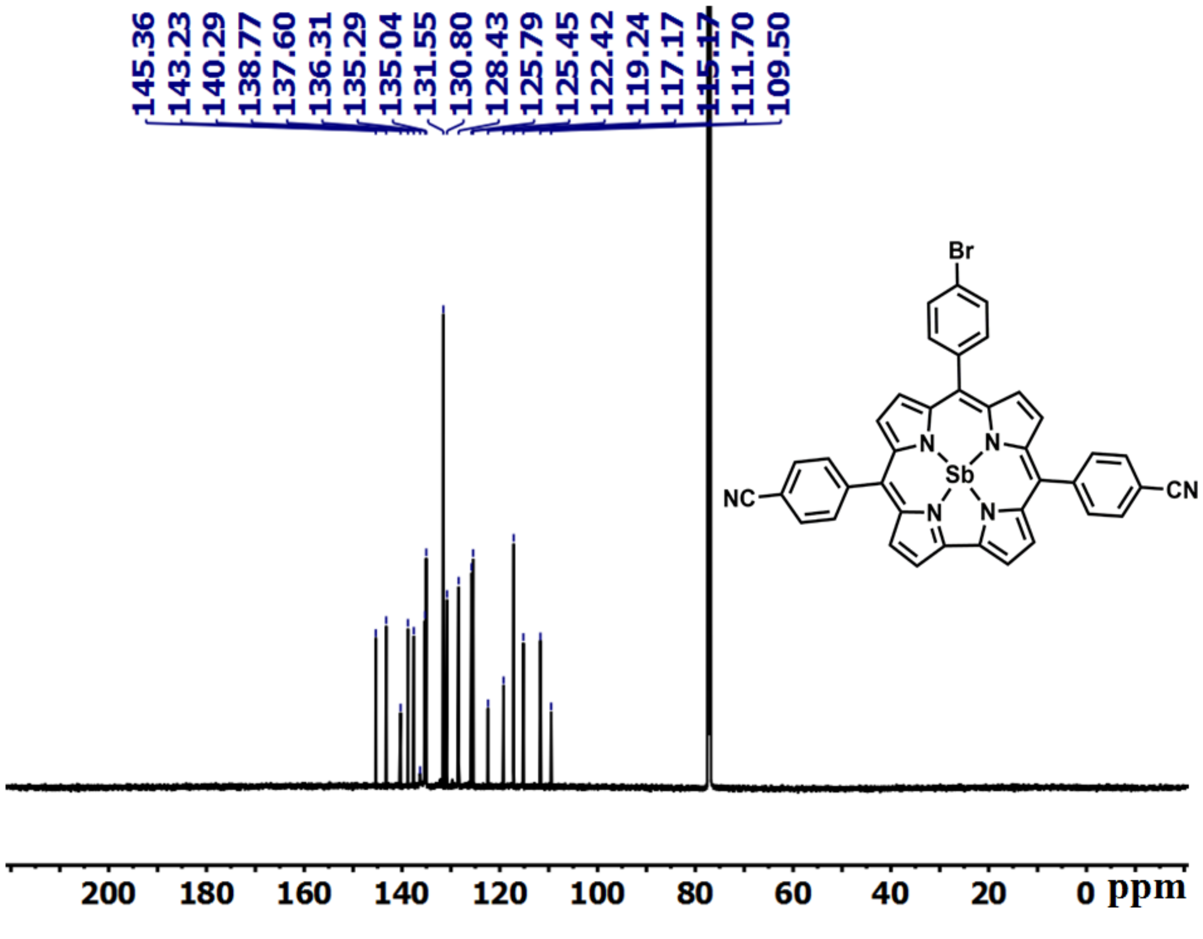

Figure S4 ^{13}C {^{1}H} NMR (176 MHz) spectrum of antimony(III) corrole, **1-H** in $CDCl_3$.

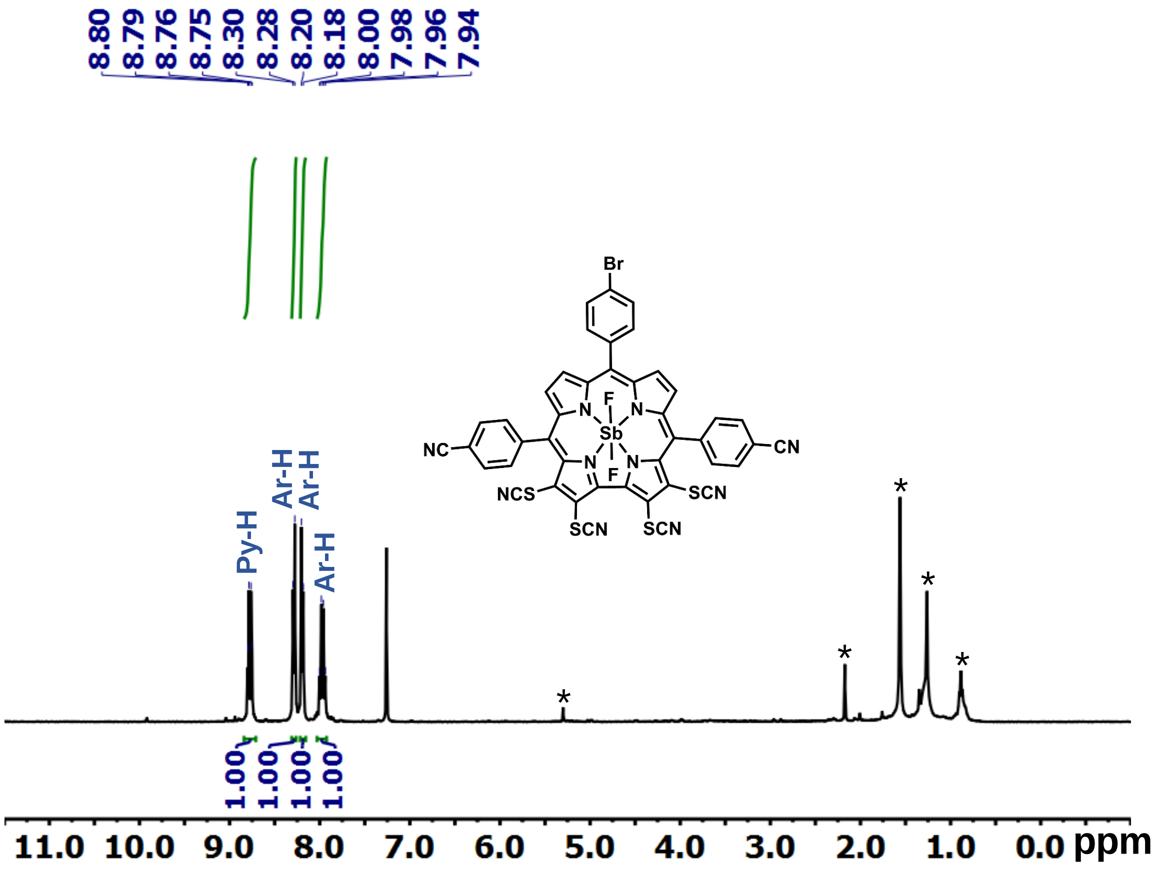

Figure S5 ^{1}H NMR (400 MHz) spectrum of antimony(V) corrole, **2-SCN** in $CDCl_3$.

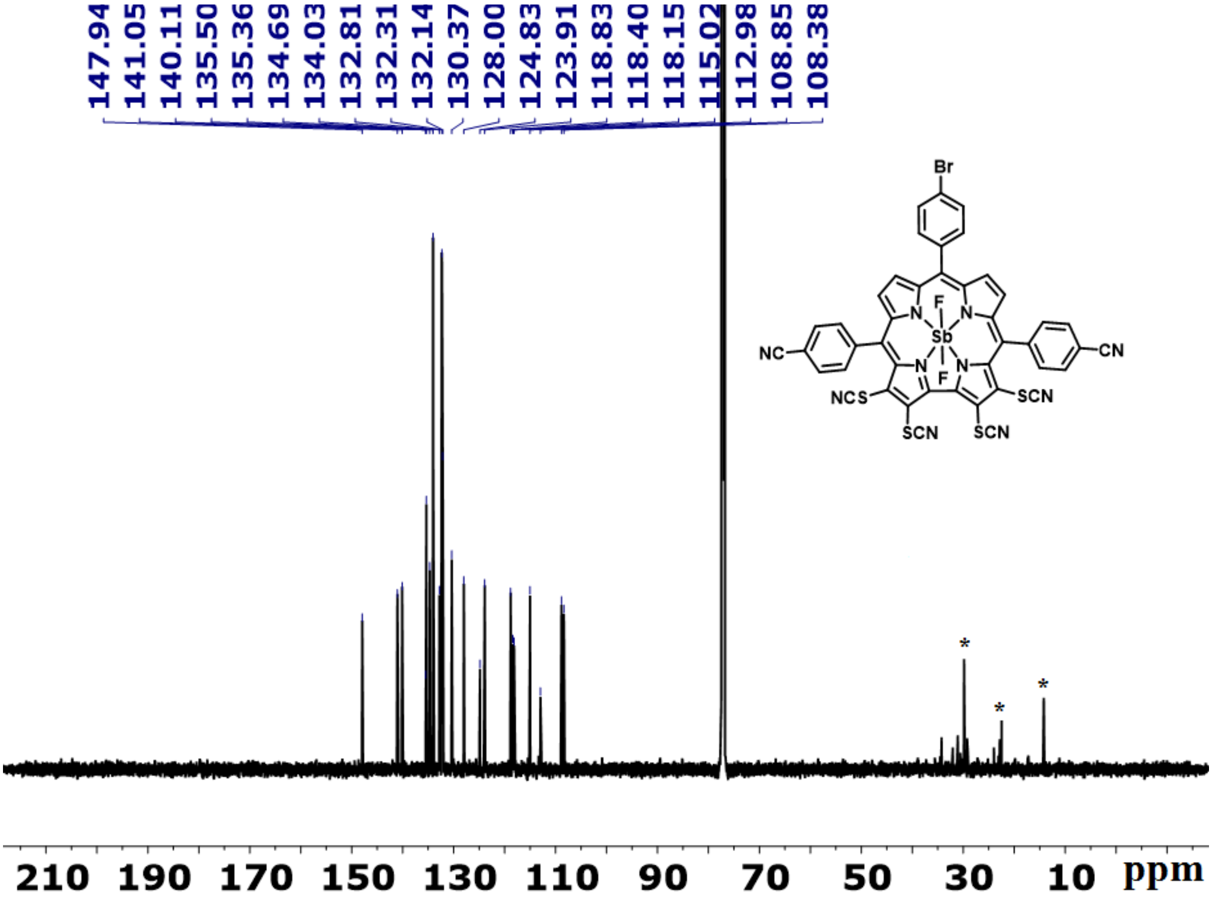

Figure S6 $^{13}C\ \{^{1}H\}$ NMR (176 MHz) spectrum of antimony(V) corrole, **2-SCN** in $CDCl_3$.

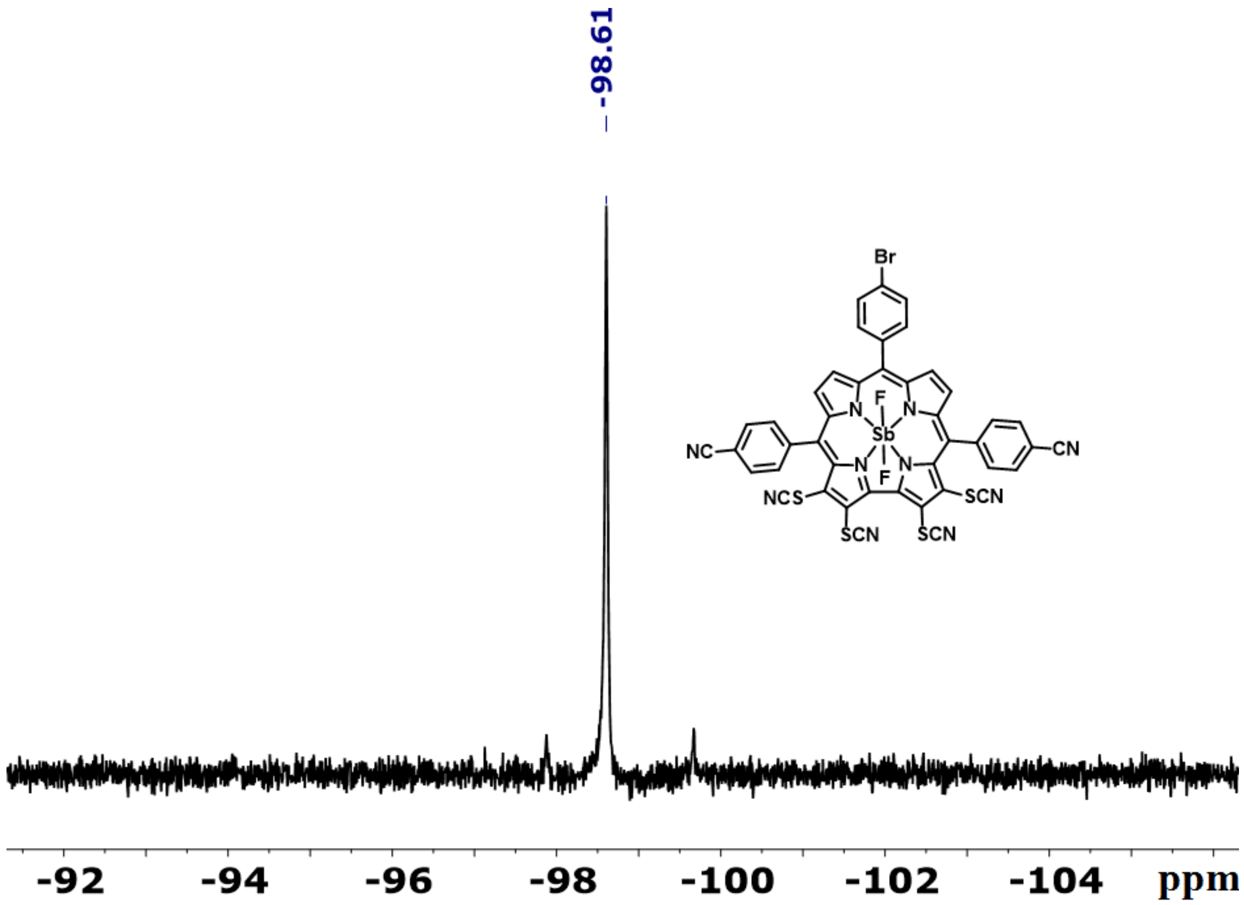

Figure S7 ^{19}F {^{1}H} NMR (377 MHz) spectrum of antimony(V) corrole, **2-SCN** in $CDCl_3$.

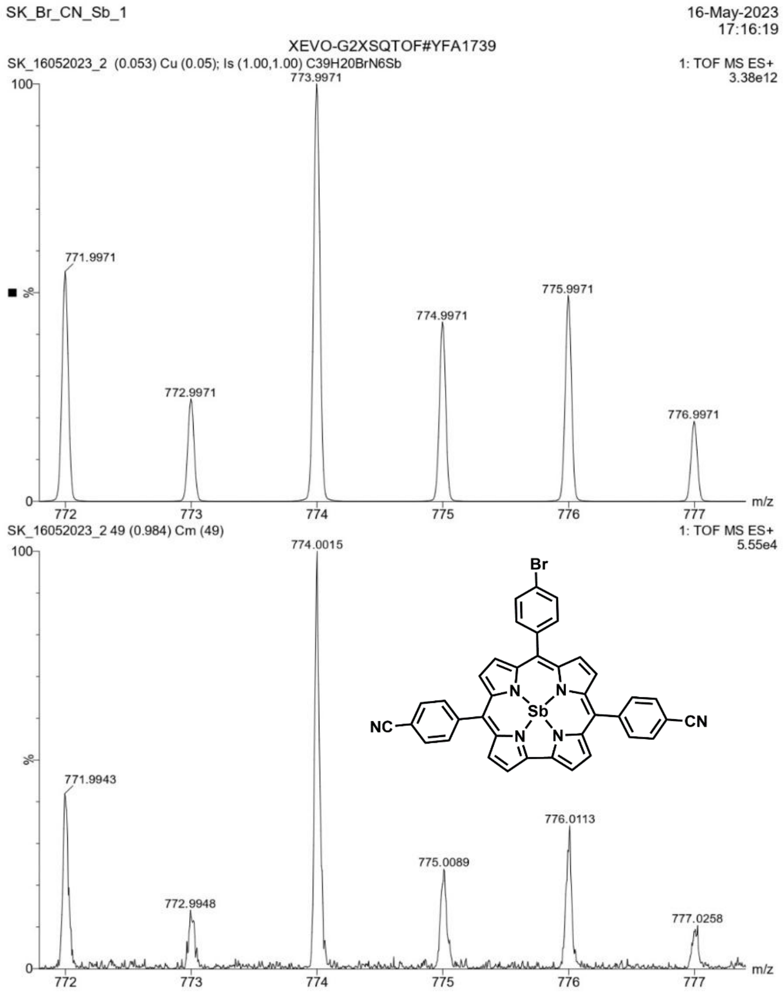

Figure S8 ESI- MS spectrum of antimony(III) corrole, **1-H** in CH_3CN shows the measured spectrum with an isotopic distribution pattern.

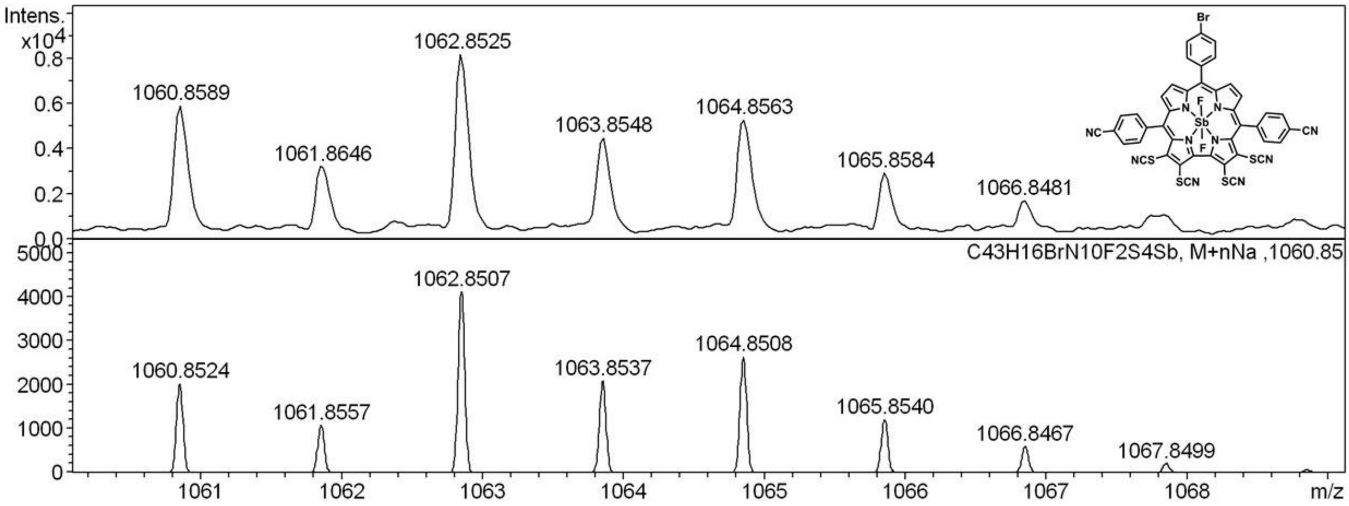

Figure S9 ESI- MS spectrum of antimony(V) corrole, **2-SCN** in CH_3CN shows the measured spectrum with an isotopic distribution pattern.

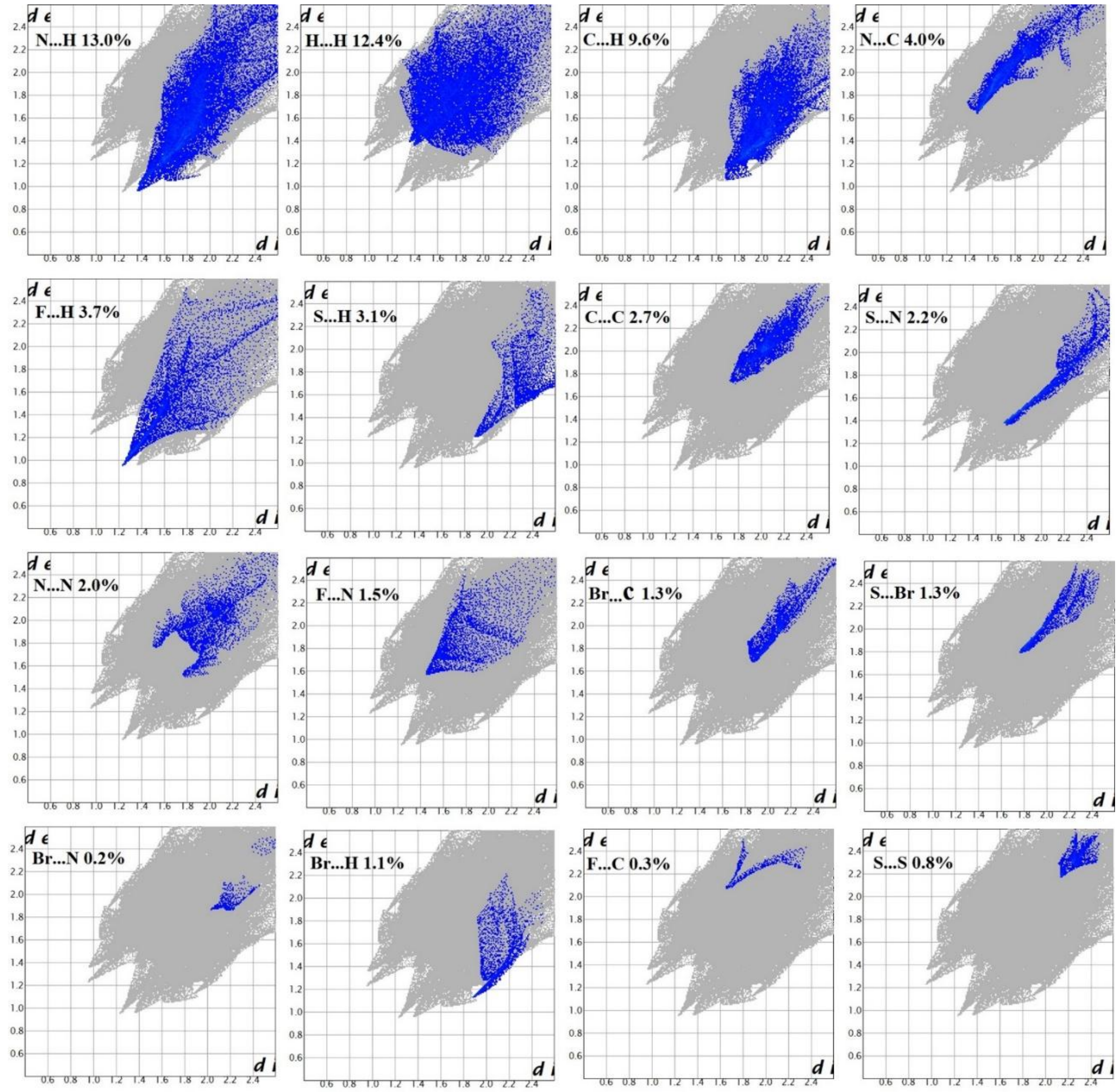

Figure S10 Fingerprint plots of antimony(V) corrole, **2-SCN** with different interactions highlighted in color.

Table S3 Contributions of various interactions in percentage to Hirshfeld surface area in antimony(V) corrole, **2-SCN.**

**Relative interactions**	**Present in 2**
N···H	13.0 %
H···H	12.4 %
C···H	9.6 %
N···C	4.0 %
F···H	3.7 %
S···H	3.1 %
C···C	2.7 %
S···N	2.2 %
N···N	2.0 %
F···N	1.5 %
Br···C	1.3 %
S···Br	1.3 %
Br···N	0.2 %
Br···H	1.1 %
F···C	0.3%
S···S	0.8%

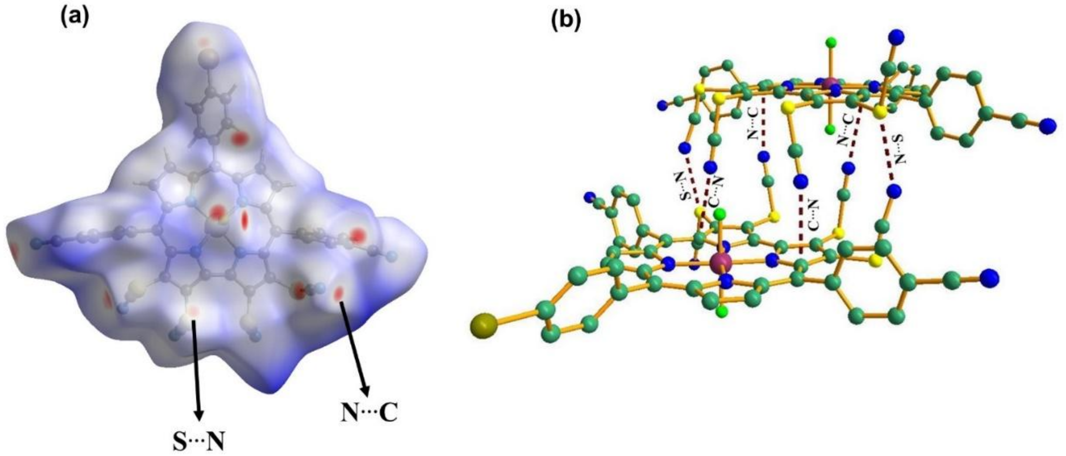

Figure S11 (a) Hirshfeld surface, and (b) supramolecular assemblies in antimony(V) corrole, **2-SCN**.

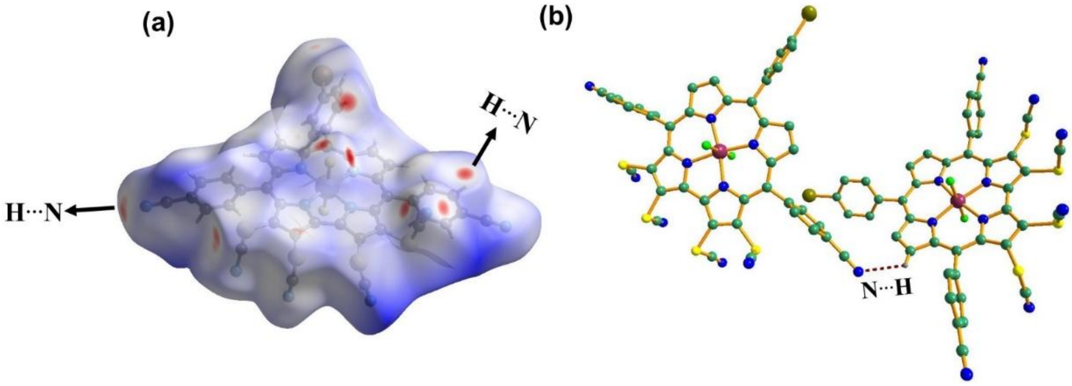

Figure S12 (a) Hirshfeld surface, and (b) hydrogen bonding interactions in antimony(V) corrole, **2-SCN**

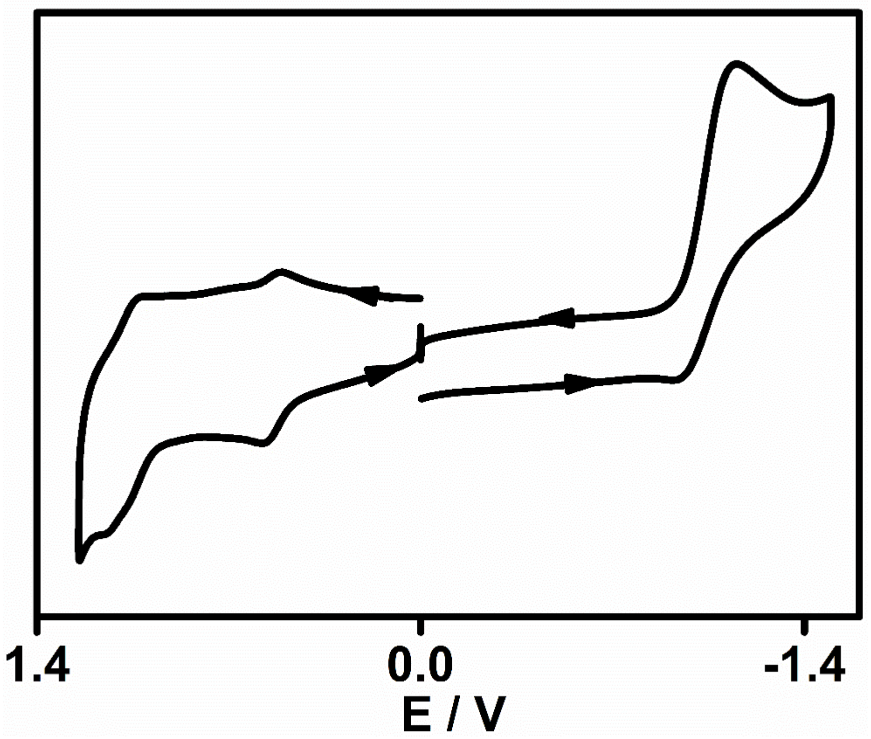

Figure S13 Cyclic voltammograms of antimony(III) corrole, **1-H** in dichloromethane. The potentials are *vs.* Ag/AgCl.

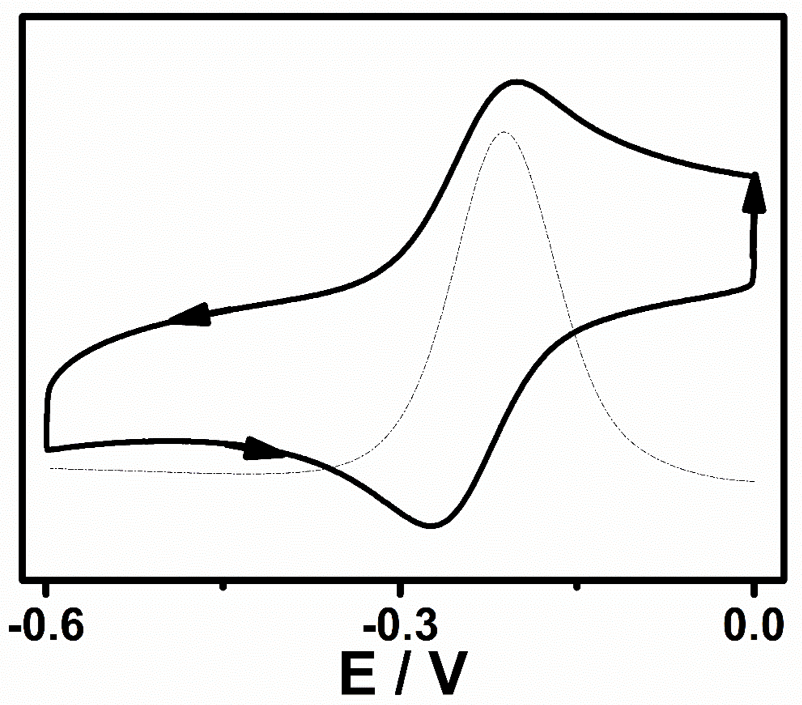

Figure S14 Cyclic voltammograms and differential pulse voltammograms of antimony(V) corrole, **2-SCN** in dichloromethane. The potentials are *vs.* Ag/AgCl.

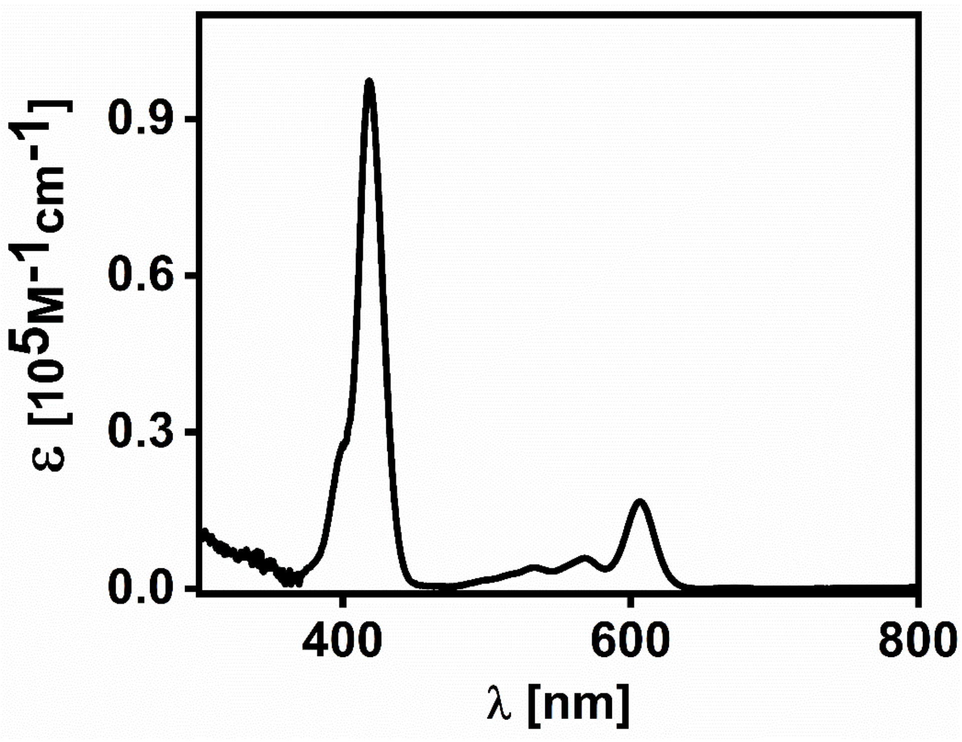

Figure S15 Electronic absorption spectrum of antimony(V) corrole, **2-H** in DCM

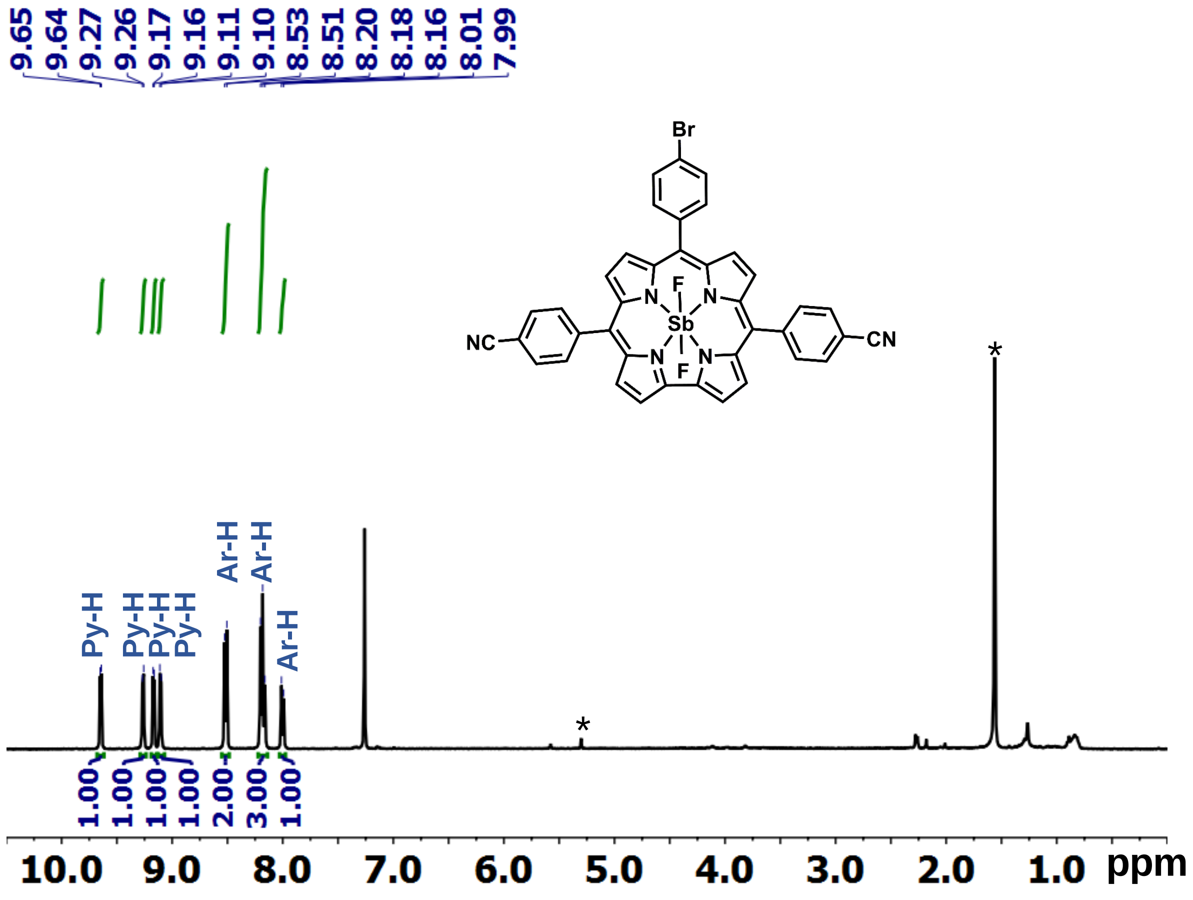

Figure S16 a) ^{1}H NMR (400 MHz) spectrum of antimony(V) corrole, **2-H** in $CDCl_3$.

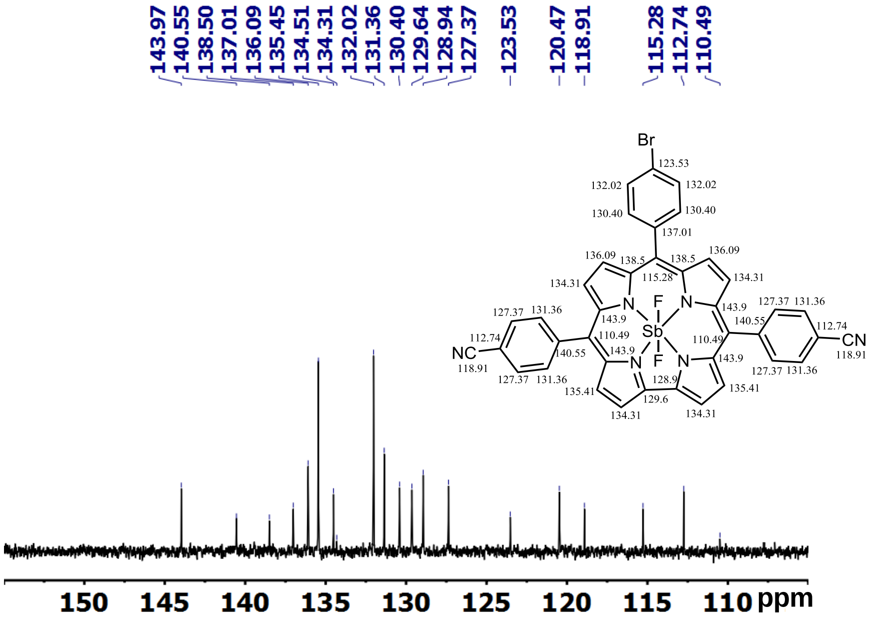

143.97
140.55
138.50
137.01
136.09
135.45
134.51
134.31
132.02
131.36
130.40
129.64
128.94
127.37
123.53
120.47
118.91
115.28
112.74
110.49
Br
NC
CN
Sb
F
N
150
145
140
135
130
125
120
115
110
ppm

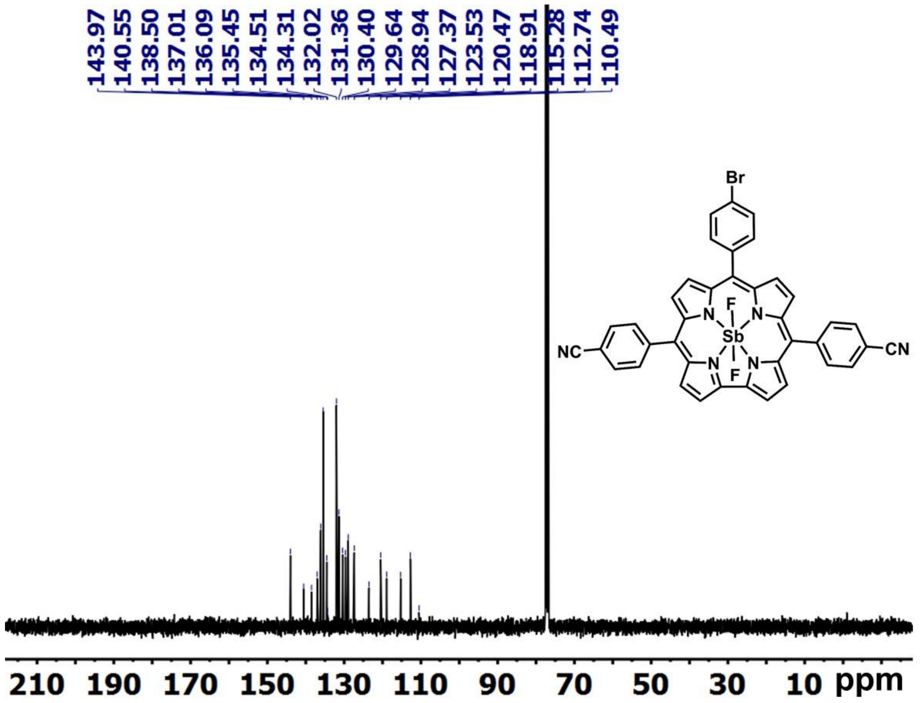

Figure S16 b) ^{13}C NMR $\{^{1}H\}$ (176 MHz) spectrum of antimony(V) corrole, **2-H** in $CDCl_3$.

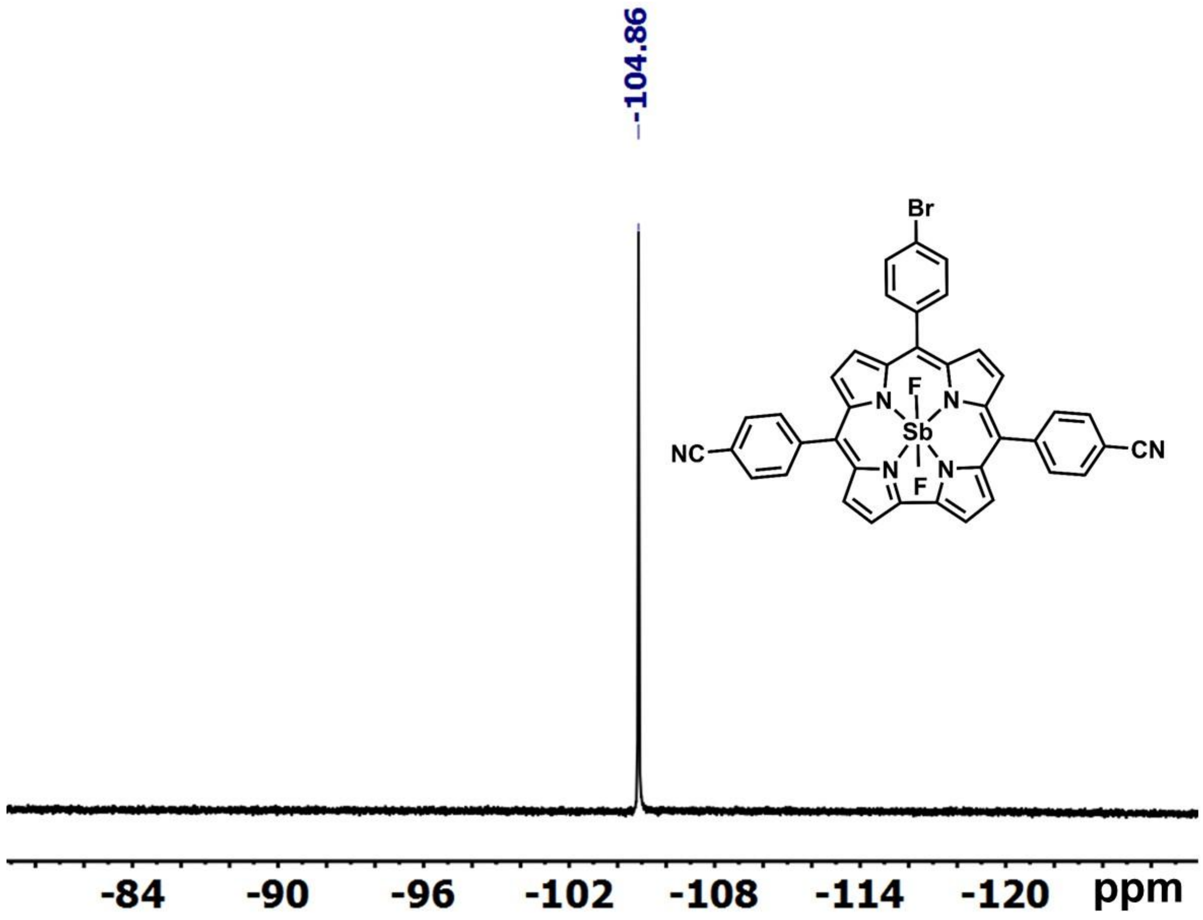

Figure S16 c) ^{19}F NMR {^{1}H} (377 MHz) spectrum of antimony(V) corrole, **2-H** in $CDCl_3$.

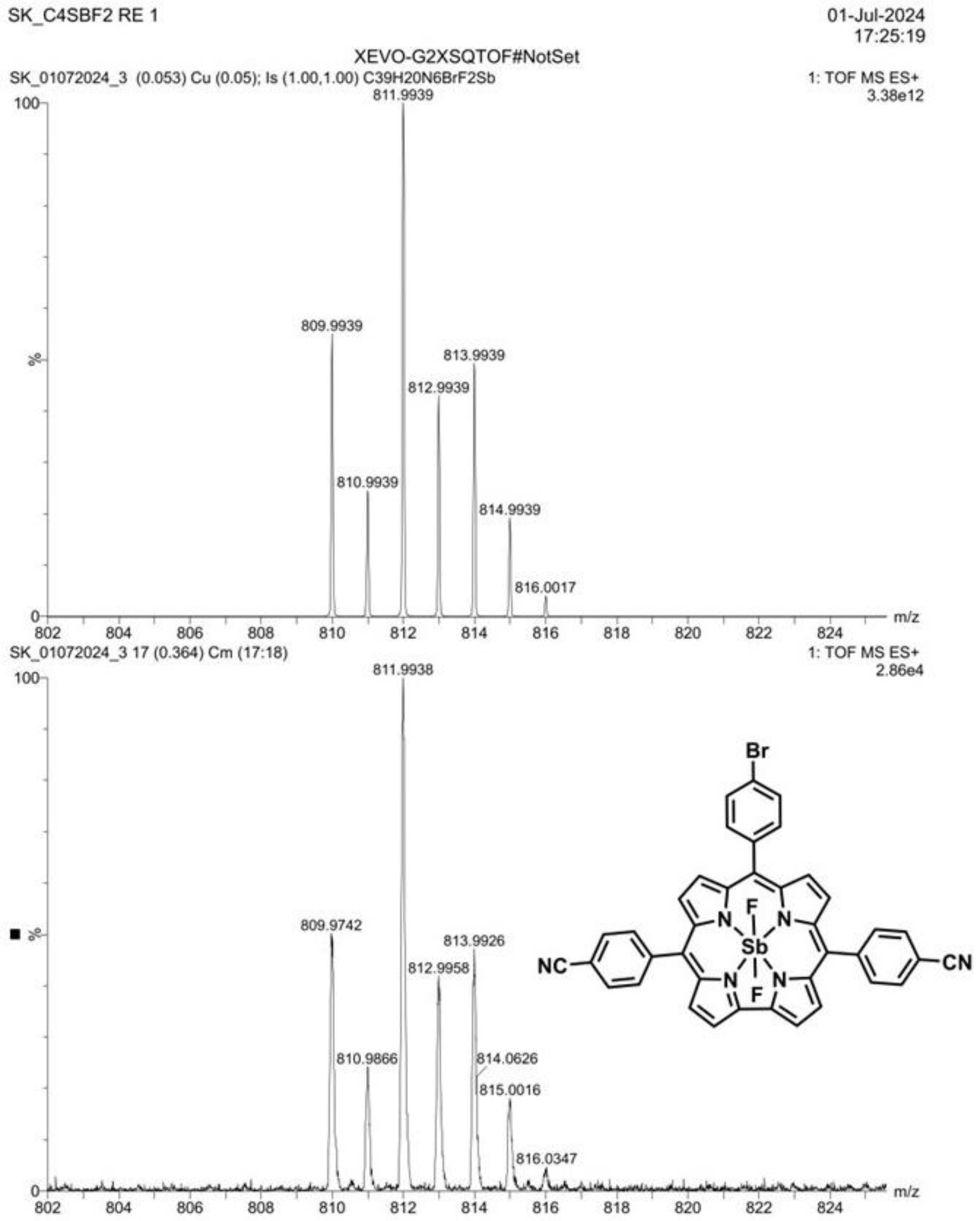

Figure S17 ESI- MS spectrum of antimony(V) corrole, **2-H** in CH_3CN shows the measured spectrum with an isotopic distribution pattern.

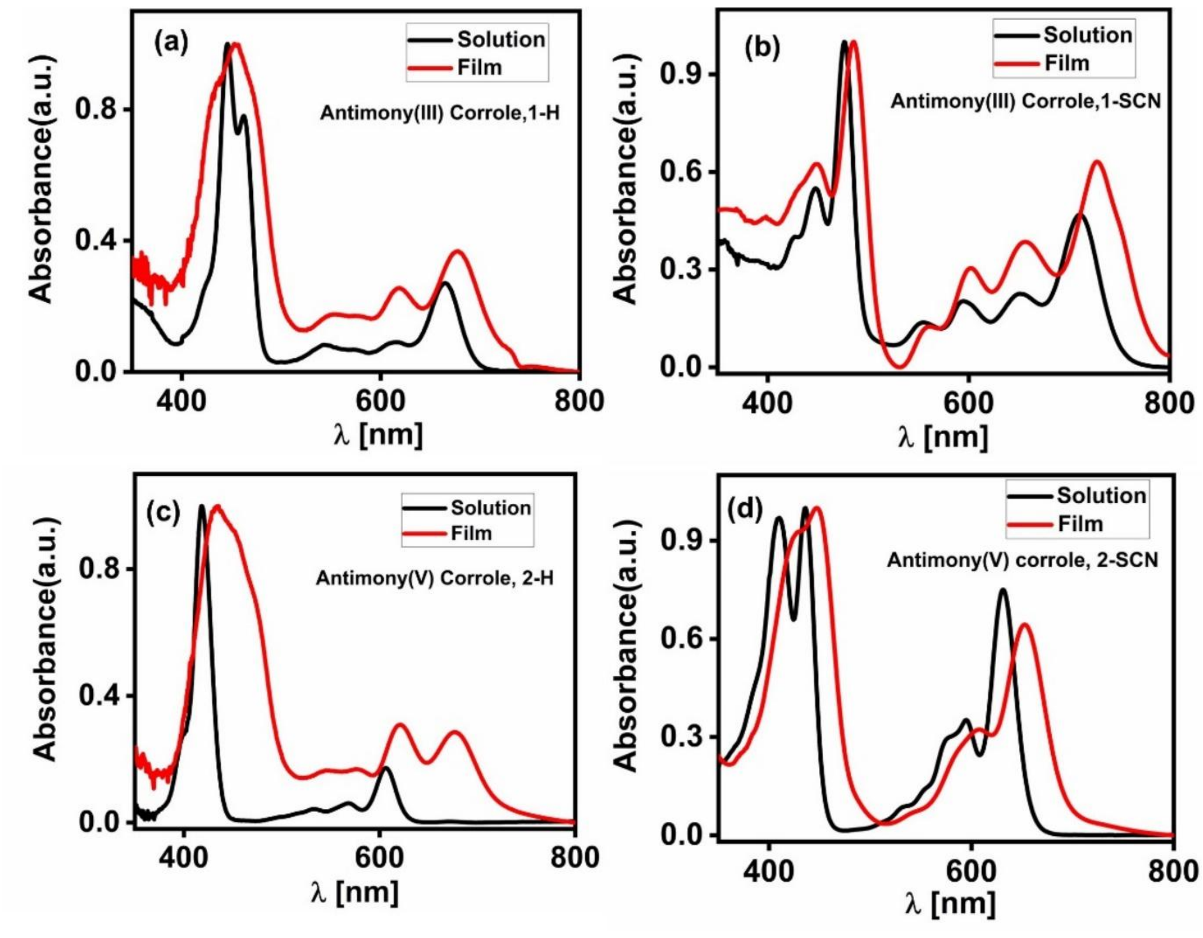

Figure S18 Electronic absorption spectra of antimony corroles in solution and thin film.

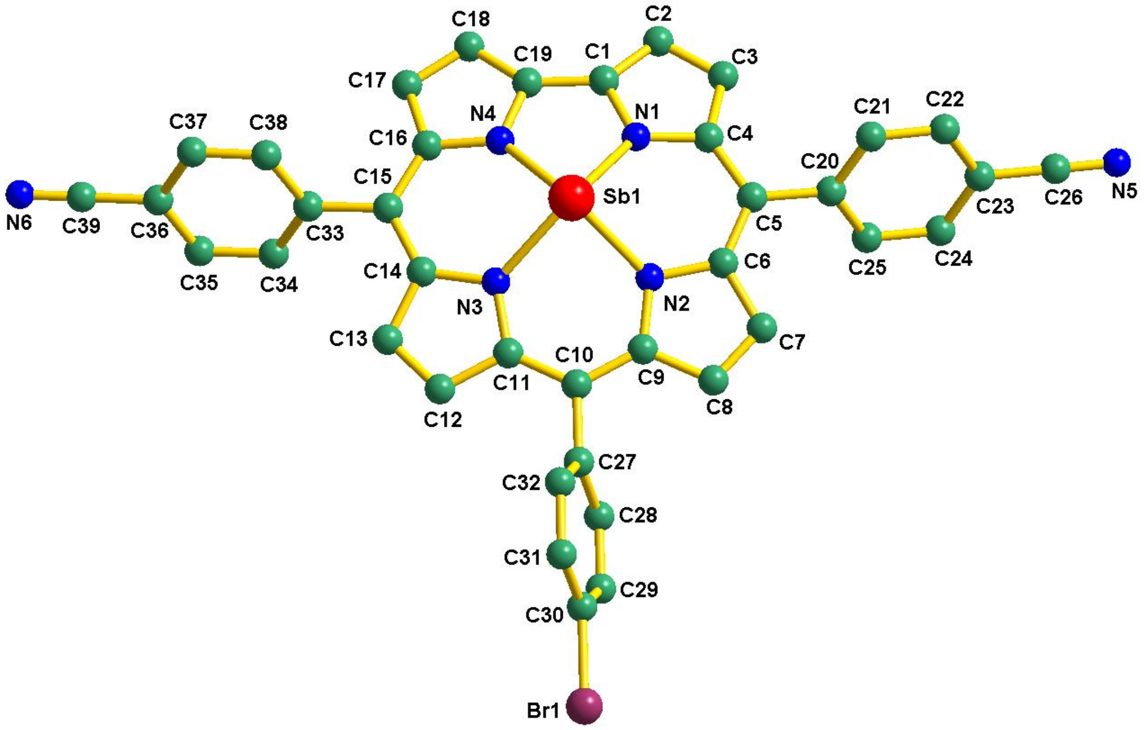

Figure S19 DFT-optimized geometry of antimony(III) corrole, **1-H** using the 6-311G (d, p) basis set.

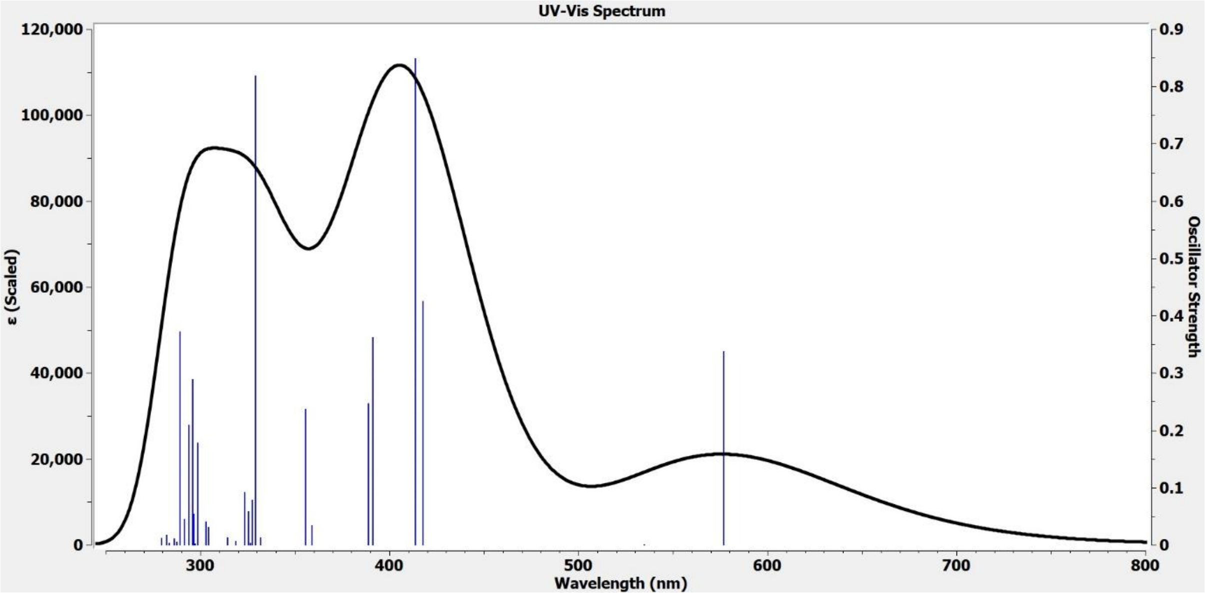

Figure S20 TD-DFT-based electronic absorption spectra of antimony(III) corrole, **1-H.**

Table S4 TD-DFT Calculated Electronic Transitions for antimony(III) corrole, **1-H**.

State	Energy (eV)	Wavelength (nm)	Oscillator Strength	Orbital contributions[a]
S1	2.14	578.60	0.3364	HOMO->LUMO (79%)<br>HOMO-1->LUMO+1 (17%)
S2	2.94	421.15	0.4244	HOMO-1->LUMO (24%)<br>HOMO->LUMO+1 (26%)<br>HOMO->LUMO+2 (40%)
S3	2.97	417.18	0.8478	HOMO-1->LUMO+1 (35%)<br>HOMO->LUMO+3 (51%)
S4	3.14	394.78	0.3615	HOMO-1->LUMO (16%)<br>HOMO->LUMO+2 (50%)
S5	3.16	392.35	0.246	HOMO-1->LUMO+1 (26%)<br>HOMO->LUMO+3 (40%)
S6	3.44	359.44	0.2357	HOMO-1->LUMO+3 (84%)

S7	3.72	333.19	0.8175	HOMO-2->LUMO (63%)
S8	4.09	302.97	0.1772	HOMO-3->LUMO+1 (71%)<br>HOMO-2->LUMO+1 (19%)
S9	4.12	300.87	0.0529	HOMO-1->LUMO+5 (78%)
S10	4.13	300.20	0.2886	HOMO->LUMO+10 (63%)
S11	4.15	298.31	0.2087	HOMO-7->LUMO (28%)<br>HOMO-5->LUMO (26%)<br>HOMO-1->LUMO+5 (17%)
S12	4.18	296.02	0.0441	HOMO-1->LUMO+6 (85%)
S13	4.22	293.56	0.3714	HOMO-7->LUMO (33%)<br>HOMO-3->LUMO+1 (17%)<br>HOMO-2->LUMO+1 (28%)

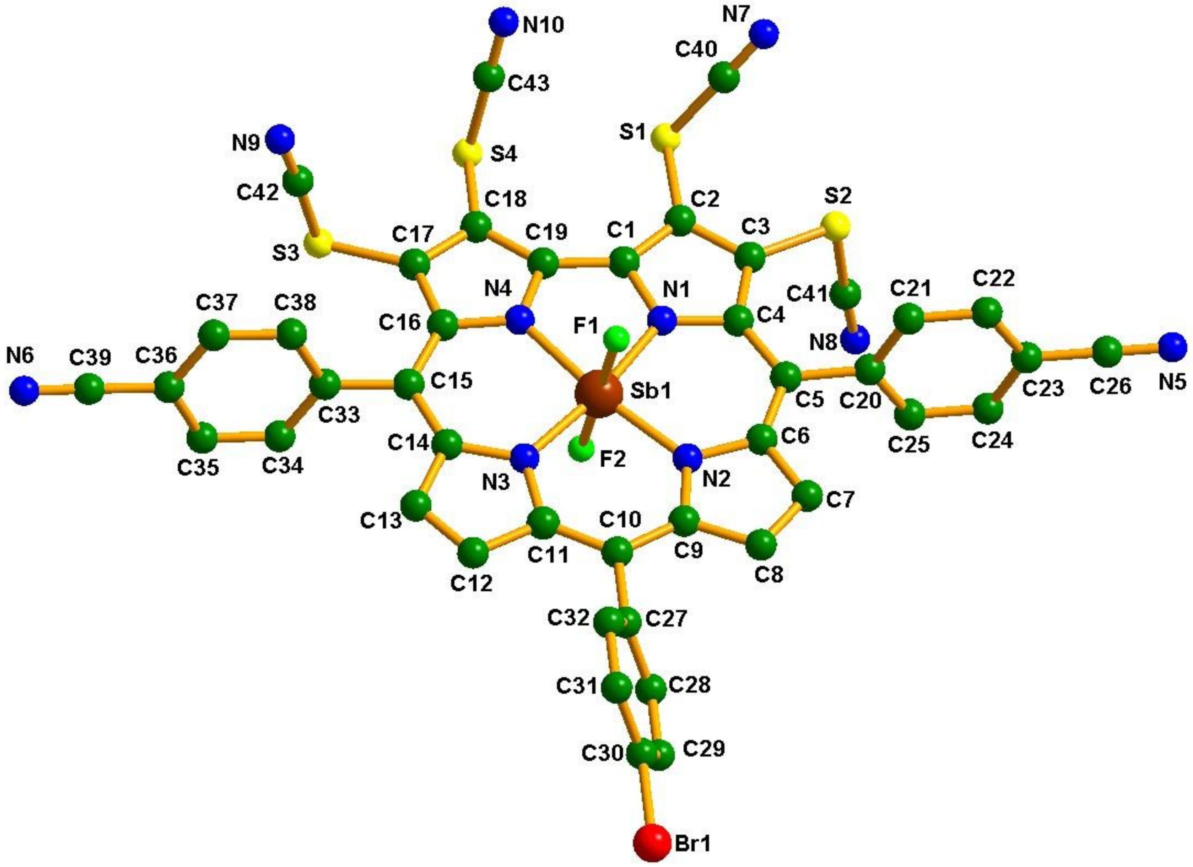

Figure S21 DFT-optimized geometry of antimony(V) corrole, **2-SCN** using the 6-311G (d, p) basis set.

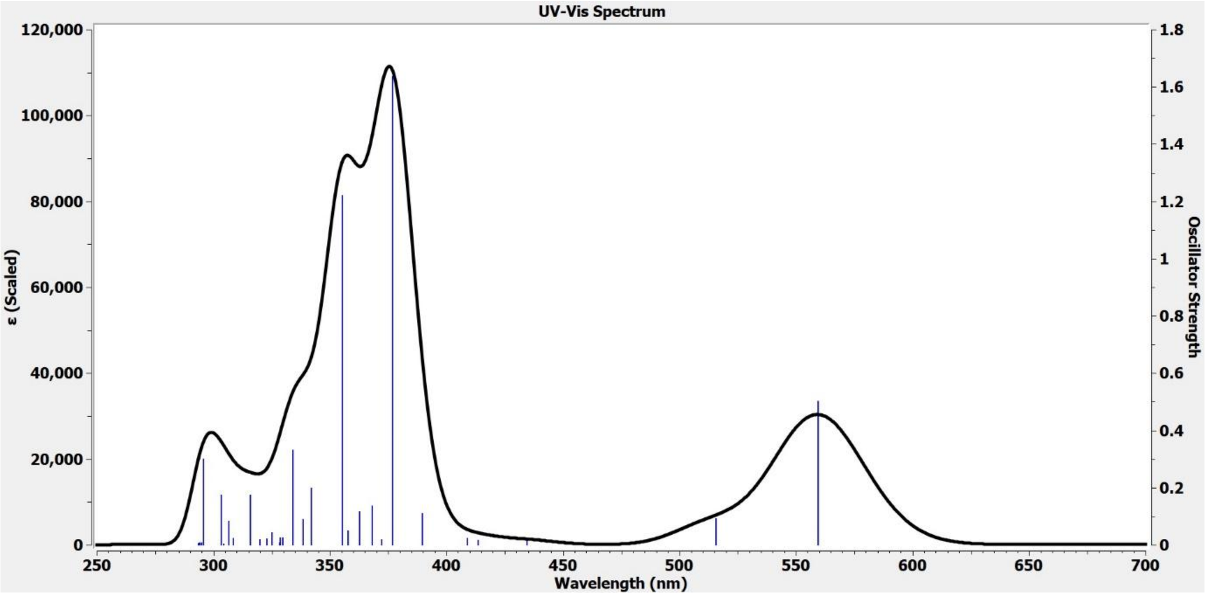

Figure S22 TD-DFT-based electronic absorption spectra of antimony(V) corrole, **2-SCN**.

Table S5 TD-DFT Calculated Electronic Transitions for antimony(V) corrole, **2-SCN**.

State	Energy (eV)	Wavelength (nm)	Oscillator Strength	Orbital contributions[a]
S1	2.21	559.54	0.5011	HOMO-1->LUMO+1 (14%)<br>HOMO->LUMO (82%)
S2	3.29	376.80	1.6353	HOMO-2->LUMO+1 (18%)<br>HOMO-1->LUMO (19%),<br>HOMO-> LUMO +1 (41%)
S3	3.36	368.23	0.1342	HOMO-7->LUMO (85%)
S4	3.41	362.72	0.1159	HOMO-8->LUMO (89%)
S5	3.48	355.28	1.2193	HOMO-1->LUMO+1 (54%)
S6	3.62	341.99	0.1977	HOMO-10->LUMO (47%)<br>HOMO-2->LUMO+1 (35%)
S7	3.71	334.12	0.3295	HOMO-11->LUMO (22%)<br>HOMO-10->LUMO (25%)<br>HOMO-2->LUMO+1 (23%)

S8	3.92	315.82	0.1736	HOMO-4->LUMO+1 (81%)
S9	4.08	303.24	0.172	HOMO-15->LUMO (61%)<br>HOMO-5->LUMO+1 (24%)
S10	4.19	295.83	0.2985	HOMO-1->LUMO+2 (85%)

Table S6 Calculated dipole (in Debye) and quadrupole moments (in Debye-Ang): The dipole and quadrupole moments (anisotropic values) are calculated by DFT, where Qπ is the out-of-plane component and is perpendicular to the molecular plane.

**Molecules**	**D (total) [Debye]**	**Q_1 (in-plane) [Debye-Ang]**	**Q_2 (in-plane) [Debye-Ang]**	**Q_π (out of plane) [Debye-Ang]**
Antimony(III) corrole, **1-H**	1.5753	-423.0151	-290.8655	-280.3598
Antimony(V) corrole, **2-SCN**	11.9936	-431.5144	-505.9071	-408.9860

Appendix-1 FLIM measurements of Antimony corroles and PVDF-HFP mixed system.

For FLIM measurements, we prepared a blend of antimony corrole complex with PVDF: HFP using N, N-Dimethylformamide (DMF) solvent at a concentration of 0.1 mg/ml and 1 mg/ml, respectively. Low concentration was utilized to ensure that aggregation-induced effects do not dominate the lifetime variation. The solutions were mixed in different volume ratios (as shown in the table below) and drop casted to obtain the films on which the FLIM measurements were then performed.

Sample	Antimony corroles, (Antimony(III) corrole, **1-H** or Antimony(V) corrole, **2-SCN)**	PVDF-HFP
Pristine	100 µL	
1:1	100 µL	100 µL
1:2	100 µL	200 µL
1:3	100 µL	300 µL

For the dielectric constant measurement, thin films were prepared in similar volume ratios. However, the concentration was increased by ten times (~ 10 mg/ml) to ensure that the capacitive leakage was minimized.

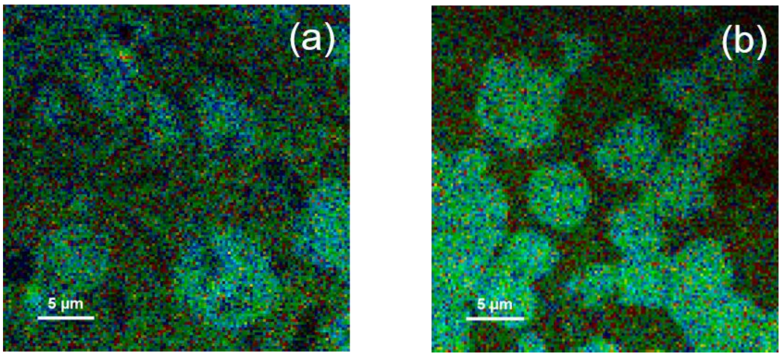

Figure S23 Fluorescence lifetime images for blended films of PVDF: HFP with antimony(V) corrole, **2-SCN** in ratio (a) 1:1 and (b) 1:2.

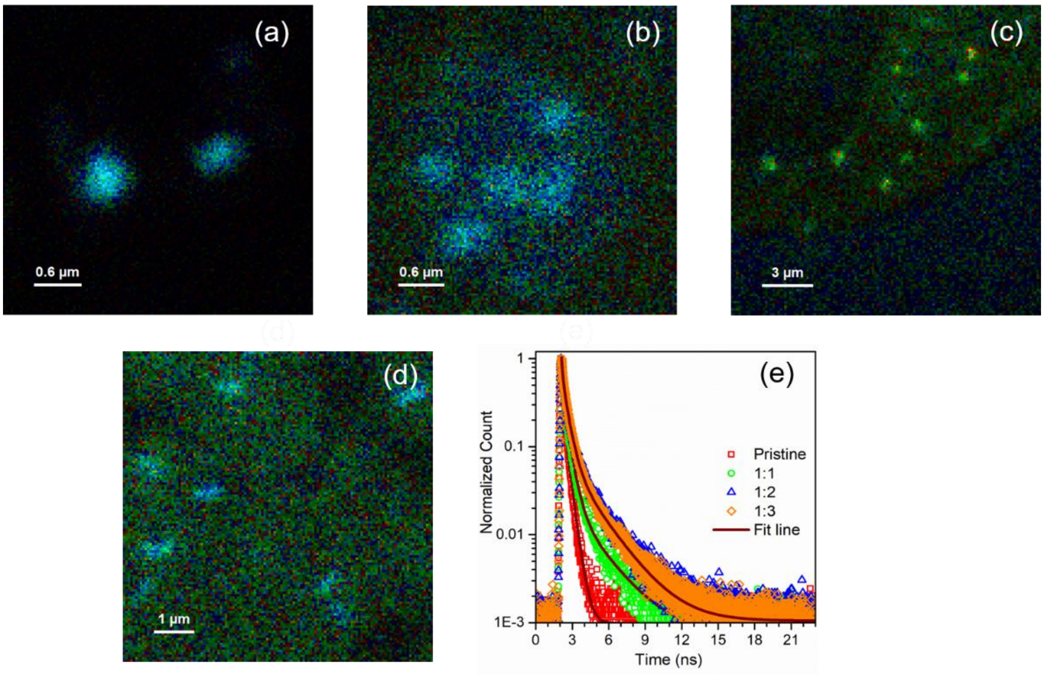

Figure S24 Fluorescence lifetime images for (b) pristine film of antimony(III) corrole, **1-H**; blended films of PVDF: HFP with antimony(III) corrole, **1-H** in ratio (c) 1:1, (d) 1:2, (e) 1:3. (f) plot of the fluorescence lifetime for different films estimated to be 476 ps, 724 ps, and 642 ps, respectively.

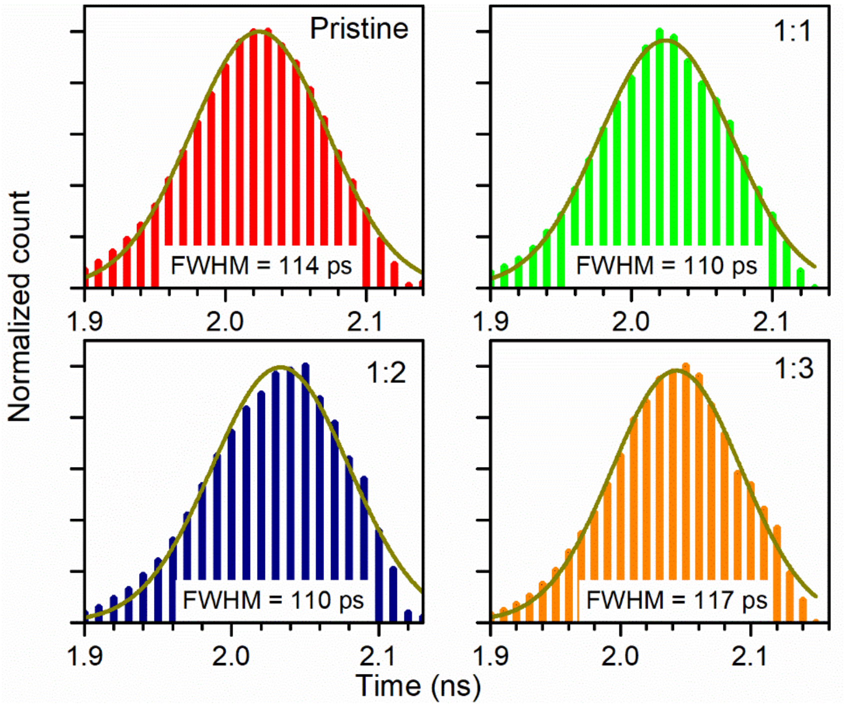

Figure S25 Fluorescence lifetime distribution (fitted with Gaussian function) or pristine antimony(III) corrole, **1-H** blended with increasing PVDF-HFP amounts.

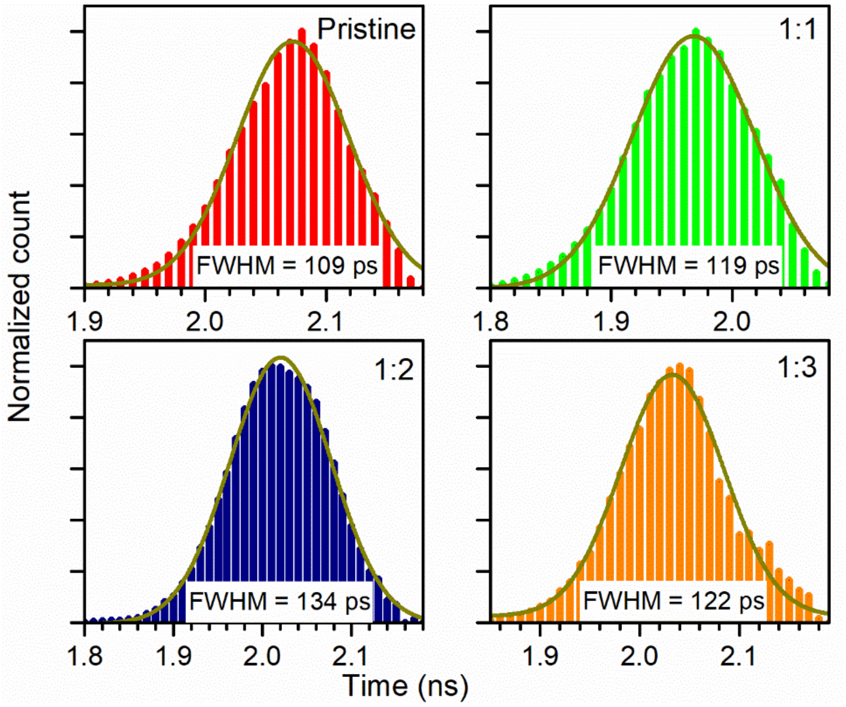

Figure S26 Fluorescence lifetime distribution (fitted with Gaussian function) for pristine antimony(V) corrole, **2-SCN** and blended with increasing PVDF-HFP amount with different ratios.

Appendix-2 Dielectric properties measurements of the thin films of silicon substrates by taking different volume ratios of both antimony corroles (Antimony (III) corrole, **1-H** and Antimony(V) corrole, **2-SCN**) and PVDF-HFP

To understand the reason behind the observed increase in a lifetime upon blending antimony(V) corrole, **2-SCN** with PVDF: HFP, we performed detailed impedance spectroscopy measurements by fabricating a sandwich device structure consisting of Si/SiO_2/ Antimony Corrole (Antimony(III) corrole, **1-H** and antimony(V) corrole, **2-SCN**): PVDF-HFP/Au. Antimony corroles (antimony(III) corrole, **1-H** or antimony(V) corrole, **2-SCN**) was dissolved in DMF with a concentration of 1 mg/mL, and blended with PVDF-HFP in DMF (10 mg/mL). This solution was drop casted and the films were annealed at 100°C for 1 hour. The device was completed by thermally evaporating Au of 25 nm thickness using a thermal evaporation technique (deposition rate ~ 0.3 Å/s and chamber pressure ~10^{-6} mbar). Volume of the solutions used in the blends is shown below:

Sample	Antimony corrole (antimony(III) corrole, **1-H** or antimony(V) corrole, **2-SCN)**	PVDF-HFP
PVDF-HFP		100 μL
1:1	100 μL	100 μL
1:2	100 μL	200 μL

It was observed that an increase in the PVDF: HFP fraction increases the dielectric constant of the films. This increase in dielectric constant can be directly correlated to the enhanced fluorescence lifetime of the corresponding solutions.

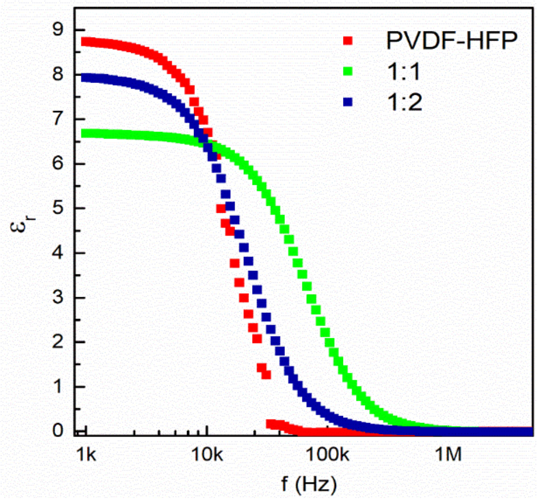

Figure S27 Frequency-dependent dielectric constant plots of PVDF-HFP and a mixture of antimony(V) corrole, **2-SCN** and PVDF-HFP in 1:1 and 1:2 ratios.

Appendix-3 Charge transport measurement

In addition to the fabrication of hole-only devices, we also performed detailed transport measurements with electron-only devices for both the antimony corrole complexes. Notably, the electron mobility was obtained to be in the range of 10^{-7} $cm^2V^{-1}s^{-1}$, which is at least three orders of magnitude lower than the hole mobility, indicating the p-type nature of the semiconductor.

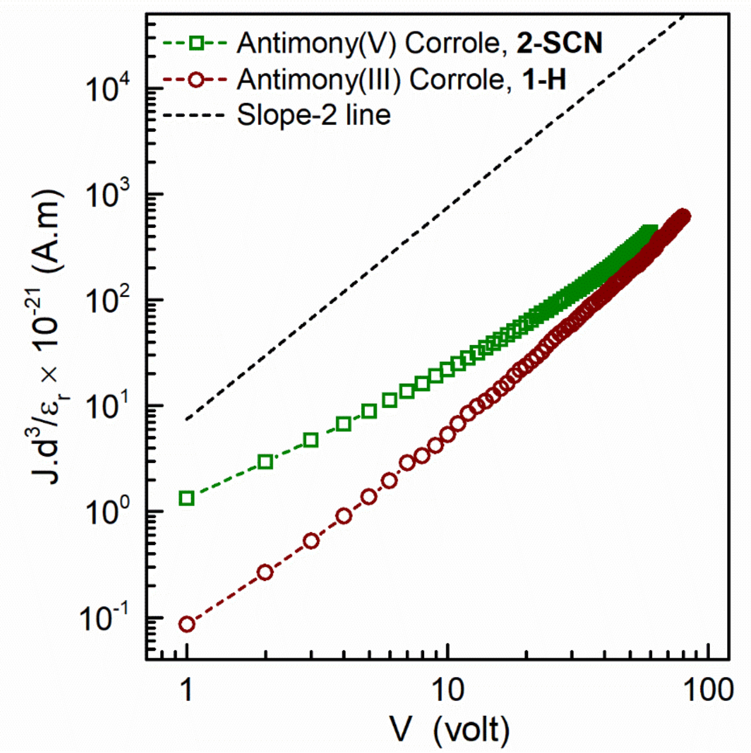

Figure S28 J-V characteristics of electron-only devices of antimony corroles (Antimony(III) corrole, **1-H** and antimony(V) corrole, **2-SCN**) (J is scaled with sample thickness (d) and dielectric constant (ε_r).

Appendix-4 XRD measurement

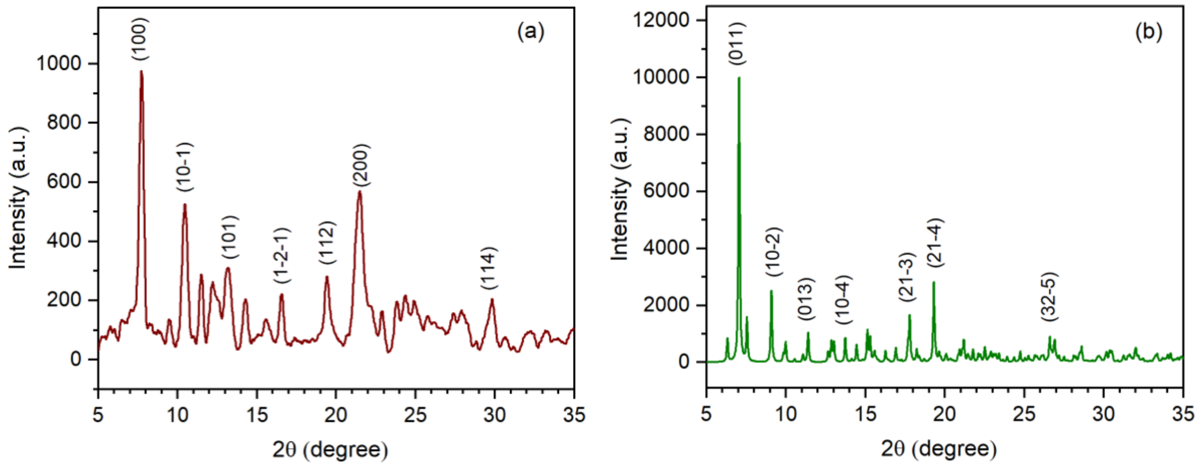

Figure S29 p-XRD pattern of antimony corroles (Antimony(III) corrole, **1-H** and Antimony(V) corrole, **2-SCN**).

In order to obtain an understanding of the charge transport trends between the different antimony corroles (Antimony(III) corrole, **1-H** and Antimony(V) corrole, **2-SCN**) we performed detailed powder XRD measurement and estimated the FWHM of the first primary peak. The FWHM of the first prominent peak increases from from 0.319° to 0.103° for antimony(V) corrole, **2-SCN** in comparison to antimony(III) corrole, **1-H** (Figure S29).

Appendix-5 Temperature dependent charge transport measurements

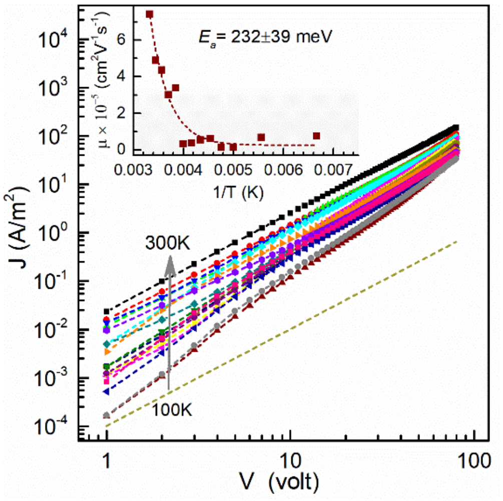

Figure S30 Temperature-dependent J-V characteristics of hole-only devices of antimony(III) corrole, **1-H** under dark conditions. Inset: Corresponding μ versus 1/T characteristics.

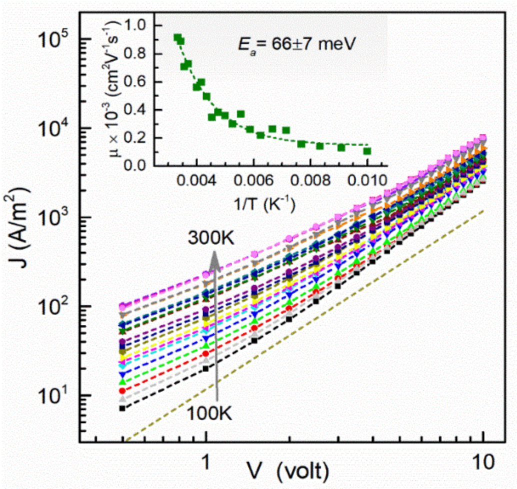

Figure S31 Temperature-dependent J-V characteristics of hole-only devices of antimony(V) corrole, **2-SCN** under dark conditions. Inset: Corresponding μ versus 1/T characteristics.

To elucidate the differences in charge transport between antimony(III) corrole, **1-H** and antimony(V) corrole, **2-SCN** , temperature-dependent *J-V* measurements of these samples were performed (shown in Figure 5c and S30). The temperature-dependent hole mobilities exhibit Arrhenius-dependence: $\mu = \mu_0 \exp(-E_a/k_B T)$, where the pre-exponential

factor μ_0 is the high-temperature limit of the mobility, E_a is the transport activation energy, k_B is the Boltzmann constant, and T is temperature. The E_a-value for antimony(III) corrole, **1-H** under dark conditions is ~232 ± 39 meV. In contrast, antimony(V) corrole, **2-SCN** devices exhibit a much lower E_a-value of ~94 ± 13 meV, indicating a lower energy barrier for hopping of charge carriers.

Upon illumination, there is a marginal increase in charge carrier mobility. At the same time, the activation energy decreases from 92 meV to 66 meV, indicating the effect of trap passivation due to photogenerated charge carriers.

Table S7: Parameters obtained from hole-only devices of different p-type polymers and antimony corroles. Average values are measured from 6 devices in each case.

Organic Semiconductor	Average hole mobility ($cm^2V^{-1}s^{-1}$)	Average Photoresponsivity at 10V	Dielectric Constant at f =1 kHz
P3HT	(4.3 ± 1.5) ×10^{-5}	(239 ±197) mA/W	3.9
PBTTT-C14	(7.9 ± 0.6) ×10^{-5}	(65 ± 55) mA/W	4.6
PCPDTBT	(4.6 ± 2.0) ×10^{-4}	No response	4.3
Antimony(III) corrole, **1-H**	(1.7 ± 0.5) ×10^{-4}	No response	2.5
Antimony(III) corrole, **1-SCN**	3.2 ± 2.2×10^{-4}	No response	7.2
Antimony(V) corrole, **2-SCN**	(3.2 ± 0.8) ×10^{-4}	(5.7 ± 3.4) A/W	8.1
Antimony(V) corrole, **2-H**	5.36 ± 0.2×10^{-6}	No response	5.1

Table S8 Device performance parameters of the single-component cells. Average parameters are obtained from 3 devices.

Organic Semiconductor	Average V_{oc} (V)	Average J_{sc} (mA/cm^2)	Average PCE (%)	Fill Factor
P3HT	0.25 ± 0.02	(2.6 ± 1.1)×10^{-2}	(2.5 ± 1.3) ×10^{-3}	38%
PCBM	0.19 ± 0.07	(2.1 ± 1.8) ×10^{-4}	(1.3 ± 1.0) ×10^{-5}	33%
Antimony(III) corrole, **1-SCN**	0.70 ± 0.03	(5.0 ± 1.6) ×10^{-3}	(6.2 ± 1.2)×10^{-4}	17%
Antimony(V) corrole, **2-SCN**	0.72 ± 0.05	(8.0 ± 2.0) ×10^{-3}	(9.9 ± 0.9)×10^{-4}	17%

Appendix-6 Solar Cell Measurement of single layer and bilayer structure

Incident photon to current conversion efficiency (IPCE) measurements were performed to correlate the cell's discrete efficiency as a function of wavelength, which clearly follows the absorption spectra (Figure S32).

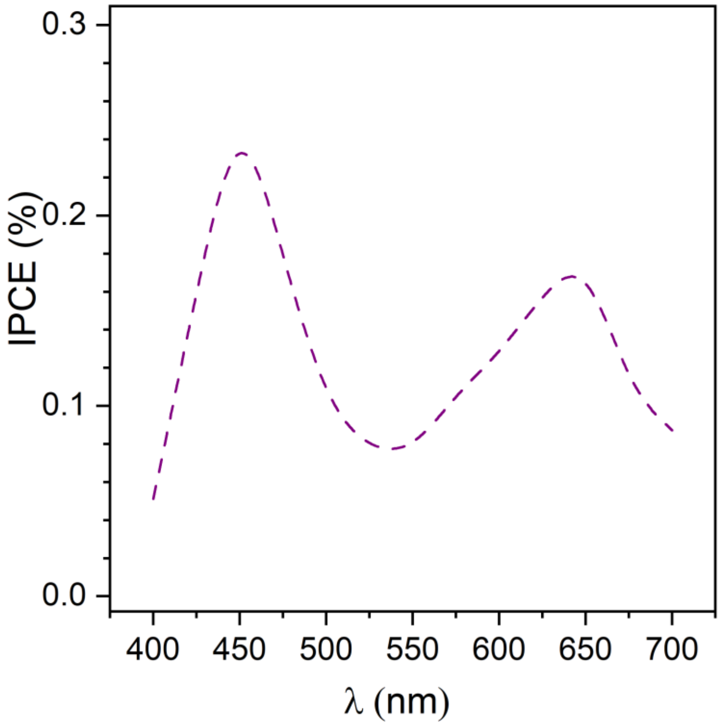

Figure S32 IPCE measurement from single component photovoltaic devices obtained from antimony(V) corrole, **2-SCN**.

photovoltaic devices.

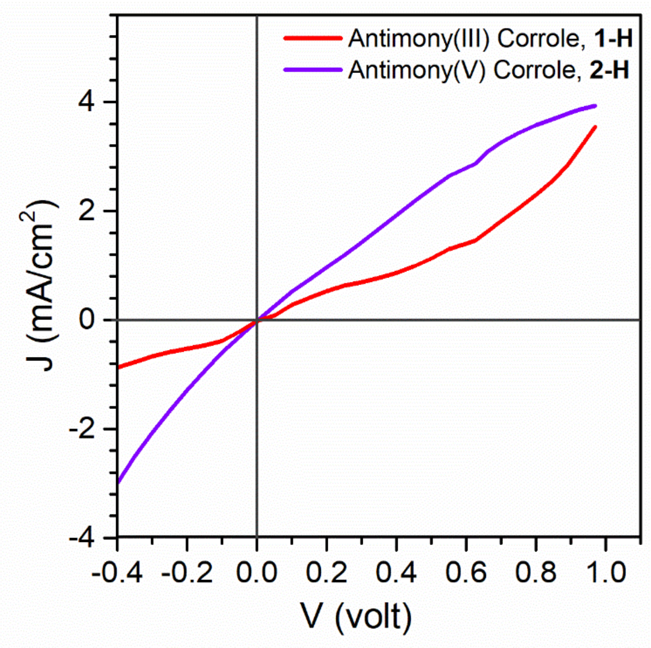

Figure S33 J–V characteristics of the single-component photovoltaic cell of antimony(III)

corrole, **1-H** and antimony(V) corrole, **2-H** under 1 sun illuminated condition.

Apart from single component solar cell measurement, we also fabricated bilayer solar cells to observe the efficacy of our designed molecule. Only antimony(V) corrole, **2-SCN** was utilized along with $PC_{61}BM$ to fabricate the following bi-layered photovoltaic device structure (fabrication details in the experimental section).

Normal: ITO/PEDOT:PSS/ antimony(V) corrole, **2-SCN**/PCBM/Al.

Inverted: ITO/ZnO/PCBM/ antimony(V) corrole, **2-SCN**/ PEDOT:PSS/Au.

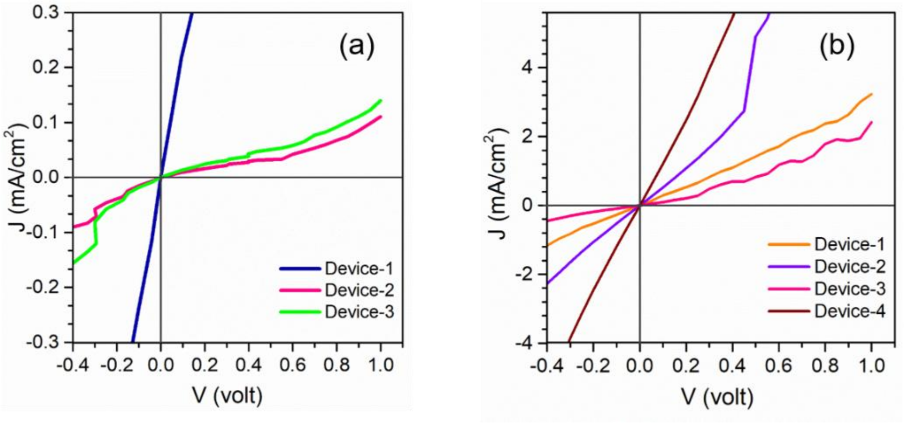

Figure S34 **(a)** J–V characteristics of the normal bilayer photovoltaic cell (multiple devices) of antimony(V) corrole, **2-SCN** under 1 sun illuminated condition. **(b)** J–V characteristics of the inverted bilayer photovoltaic cell (multiple devices) of antimony(V) corrole, **2-SCN**. All the curves are shown here were taken under 1 sun illuminated condition.

Figure S34 shows the J-V characteristics measured on multiple normal and inverted bilayer solar cell devices. For all the cases, no appreciable V_{oc} was observed which is majorly attributed to the absence of the energy offset between antimony(V) corrole, **2-SCN** and $PC_{61}BM$ (Figure S35) thereby minimizing the possibility of charge separation and V_{oc}.

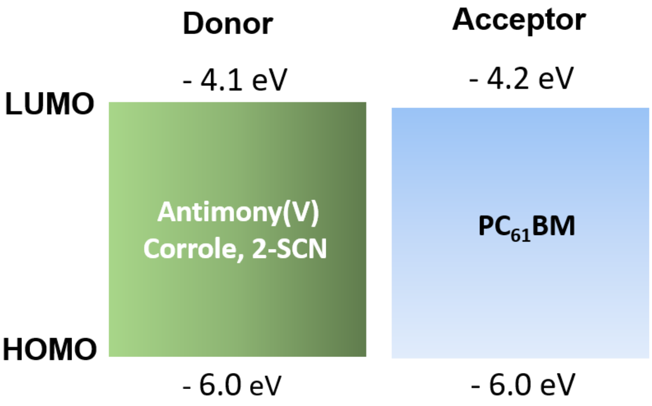

Figure S35 Energy level diagram of antimony(V) corrole, **2-SCN** as donor and $PC_{61}BM$ as acceptor.

Appendix-7 Optimized Cartesian Co-ordinates of antimony(III) corrole, **1-H**.

Cartesian coordinates of the optimized structure (in Å). antimony(III) corrole, **1-H** was optimized at the B3LYP level of theory and 6-311G (d, p) basis set. The LANL2DZ pseudopotential was used for the Sb atom.

Antimony(III) corrole, **1-H** (E = -4406.68 hartrees)

C	-3.34300	-1.66200	0.00100
C	-0.02400	1.98100	-0.11500
C	3.41000	-1.55500	-0.00200
C	-0.63800	-4.00100	0.04900
C	-1.67000	-4.88500	0.44600
C	-2.85700	-4.17100	0.39800
C	-2.55400	-2.83700	-0.02000
N	-1.20900	-2.79300	-0.26100
C	-2.78100	-0.37200	-0.04900
C	-3.47500	0.87300	0.10000
C	-2.55500	1.88600	0.08300
C	-1.26000	1.30400	-0.09800
N	-1.42200	-0.06700	-0.18900
C	1.23400	1.34500	-0.09900
C	2.51600	1.97000	0.02000
C	3.46500	0.98500	0.04300
C	2.80600	-0.28300	-0.05700
N	1.43400	-0.02300	-0.16600
C	2.66000	-2.75500	-0.01200
C	3.00400	-4.07800	0.40800

C	1.84000	-4.83000	0.45300
C	0.78100	-3.97900	0.05400
N	1.31400	-2.75300	-0.25300
C	-4.81000	-1.82500	0.19400
C	-5.46500	-1.30400	1.32000
C	-6.83000	-1.48300	1.50100
C	-7.57600	-2.19500	0.55300
C	-6.93200	-2.72900	-0.57200
C	-5.56700	-2.54600	-0.74200
C	-0.05700	3.47600	-0.05900
C	0.29700	4.16300	1.10800
C	0.26200	5.55500	1.16700
C	-0.13700	6.26900	0.04200
C	-0.49900	5.61600	-1.13100
C	-0.45700	4.22500	-1.17300
C	4.88300	-1.66700	0.17800
C	5.65300	-2.38800	-0.74800
C	7.02500	-2.52500	-0.58900
C	7.66400	-1.94300	0.51500
C	6.90500	-1.22900	1.45400
C	5.53400	-1.09800	1.28400
Br	-0.19000	8.18500	0.11100
Sb	0.03200	-1.30700	-1.18000
C	-8.98300	-2.38100	0.73400
N	-10.11900	-2.53100	0.88000
C	9.07700	-2.08100	0.68500
N	10.21900	-2.19200	0.82300
H	-1.54200	-5.90800	0.76600
H	-3.83300	-4.52400	0.69400
H	-4.54000	0.97400	0.23100
H	-2.74500	2.93900	0.21600

H	2.68200	3.03200	0.10000
H	4.53100	1.12100	0.13400
H	3.99000	-4.40000	0.70600
H	1.74400	-5.85600	0.77100
H	-4.89200	-0.77400	2.07100
H	-7.32200	-1.08300	2.37900
H	-7.50700	-3.27900	-1.30700
H	-5.07700	-2.95400	-1.61800
H	0.59600	3.60300	1.98600
H	0.53400	6.07300	2.07700
H	-0.80500	6.18200	-2.00100
H	-0.73300	3.71300	-2.08800
H	5.16700	-2.83400	-1.60700
H	7.60800	-3.07600	-1.31600
H	7.39400	-0.79200	2.31600
H	4.95300	-0.56600	2.02700

Appendix-8 Optimized Cartesian Co-ordinates of antimony(V) corrole, **2-SCN**.

Cartesian coordinates of the optimized structure (in Å). antimony(V) corrole, **2-SCN** was optimized at the B3LYP level of theory and 6-311G (d, p) basis set. The LANL2DZ pseudopotential was used for the Sb atom.

Antimony(V) corrole, **2-SCN** (E = -6564.80 hartrees)

Sb	0.01310	0.03899	-0.04768
Br	-9.06149	-2.88028	0.19888
S	4.63679	3.37459	-0.65267
S	4.87717	-3.28108	-0.35907
S	5.74921	0.03241	-0.29057
S	2.01831	5.53194	-0.64816
F	0.01898	0.11154	-1.94261
F	0.00436	-0.02800	1.84367
N	-0.80224	-1.76975	-0.11134
N	-1.70134	1.03581	-0.03794
N	1.86949	-0.71493	-0.08983
N	1.09022	1.73457	-0.01449
C	2.89742	0.19579	-0.13798
C	-2.93850	0.42481	-0.02386
C	-3.90094	1.49719	-0.04638
H	-4.97188	1.36125	-0.05073
C	2.27692	-2.02270	-0.10839
C	-0.07746	-2.95895	-0.03360
C	2.68736	-7.15585	0.42800

C	-3.16189	-0.97940	-0.00474
N	3.46542	-9.61527	0.73355
C	0.65791	3.03117	-0.13501
C	-1.16319	4.79518	-0.14546
C	1.32706	-3.10074	-0.04124
C	-0.74531	3.35646	-0.11593
C	4.08156	-0.58383	-0.20555
C	1.82861	-4.50656	0.10346
C	1.86111	3.79042	-0.31017
C	-2.16169	-1.99109	-0.02025
C	-1.80579	2.42493	-0.07343
C	2.24938	-6.70691	-0.82808
H	2.25250	-7.38565	-1.67394
C	2.45107	1.58282	-0.14139
C	3.70385	-1.94705	-0.21814
C	-4.58462	-1.43815	0.04691
C	-1.07399	-3.99553	0.08600
H	-0.85201	-5.04926	0.16470
C	2.95824	2.89488	-0.31409
C	-3.22515	2.68980	-0.06443
H	-3.66220	3.67684	-0.07770
C	-2.31790	-3.41899	0.09190
H	-3.26581	-3.92810	0.18023
C	1.81866	-5.39187	-0.98373
H	1.49427	-5.04443	-1.95893
C	-6.71978	-1.57907	1.21047
H	-7.33426	-1.36105	2.07652
C	5.30874	3.34072	0.91659
C	-6.46693	-2.59505	-0.97845
H	-6.89029	-3.14884	-1.80863
C	3.12031	-8.51328	0.59555

C	-7.24762	-2.29763	0.13815
C	2.26696	-4.96241	1.35672
H	2.27017	-4.28326	2.20362
C	-5.14159	-2.16361	-1.01808
H	-4.53632	-2.38131	-1.89295
C	-5.39252	-1.15457	1.15949
H	-4.97537	-0.60828	2.00012
N	4.41394	-4.10694	-3.07171
C	4.58672	-3.74151	-1.97801
C	2.69350	-6.27508	1.52334
H	3.02926	-6.62503	2.49348
N	5.83760	3.32062	1.95500
C	-2.01831	7.46671	-0.18002
C	6.40487	-0.70666	1.10224
C	-1.71461	5.34965	-1.31131
H	-1.80151	4.74012	-2.20535
N	6.90289	-1.20536	2.03072
C	-1.05504	5.58980	1.00534
H	-0.63349	5.17149	1.91336
C	-2.13661	6.67488	-1.33468
H	-2.55559	7.10270	-2.23915
C	-2.45927	8.83161	-0.20050
C	-1.47492	6.91641	0.99245
H	-1.38053	7.52786	1.88302
N	2.23827	6.64605	1.99010
C	2.16107	6.15878	0.93375
N	-2.82314	9.93601	-0.22169
